# Estimation of biological heart age using cardiovascular magnetic resonance radiomics

**DOI:** 10.1038/s41598-022-16639-9

**Published:** 2022-07-27

**Authors:** Zahra Raisi-Estabragh, Ahmed Salih, Polyxeni Gkontra, Angélica Atehortúa, Petia Radeva, Ilaria Boscolo Galazzo, Gloria Menegaz, Nicholas C. Harvey, Karim Lekadir, Steffen E. Petersen

**Affiliations:** 1 William Harvey Research Institute, NIHR Barts Biomedical Research Centre, Queen Mary University of London, Charterhouse Square, London, EC1M 6BQ UK; 2 Barts Heart Centre, St Bartholomew’s Hospital, Barts Health NHS Trust, West Smithfield, London, EC1A 7BE UK; 3 Department of Computer Science, University of Verona, 37134 Verona, Italy; 4 Dept. de Matematiques I Informatica, University of Barcelona, 95P7+JH Barcelona, Spain; 5 MRC Lifecourse Epidemiology Centre, University of Southampton, Southampton, UK; 6 NIHR Southampton Biomedical Research Centre, University of Southampton and University Hospital Southampton NHS Foundation Trust, Southampton, UK; 7 Health Data Research UK, London, UK; 8 Alan Turing Institute, London, UK

**Keywords:** Biomarkers, Cardiology

## Abstract

We developed a novel interpretable biological heart age estimation model using cardiovascular magnetic resonance radiomics measures of ventricular shape and myocardial character. We included 29,996 UK Biobank participants without cardiovascular disease. Images were segmented using an automated analysis pipeline. We extracted 254 radiomics features from the left ventricle, right ventricle, and myocardium of each study. We then used Bayesian ridge regression with tenfold cross-validation to develop a heart age estimation model using the radiomics features as the model input and chronological age as the model output. We examined associations of radiomics features with heart age in men and women, observing sex-differential patterns. We subtracted actual age from model estimated heart age to calculate a “heart age delta”, which we considered as a measure of heart aging. We performed a phenome-wide association study of 701 exposures with heart age delta. The strongest correlates of heart aging were measures of obesity, adverse serum lipid markers, hypertension, diabetes, heart rate, income, multimorbidity, musculoskeletal health, and respiratory health. This technique provides a new method for phenotypic assessment relating to cardiovascular aging; further studies are required to assess whether it provides incremental risk information over current approaches.

## Introduction

People around the world are living longer^1^. As such, there is increasing burden from chronic non-communicable disease of older age. Of these, cardiovascular diseases are the most common cause of death and disability in the world. Better understanding of the determinants of aging and improved methods of risk assessment are critical to promotion of cardiovascular health in older age^2^.

There are distinct age-related changes in cardiovascular structure and function, which are detectable by cardiovascular imaging^3–5^. Thus, image-derived cardiovascular phenotypes may be used to estimate biological heart age. This information may be used to investigate drivers of cardiovascular aging and to better capture individual-level risk.

Cardiovascular magnetic resonance (CMR) is the reference modality for assessment of cardiovascular structure and function. To the best of our knowledge, there are no existing models for heart age estimation using conventional CMR metrics. It is likely that such models would be hampered by high inter-correlation of conventional metrics. There is limited data on heart age models developed using whole medical images as the model input^6^. However, these models have limited interpretability, because it is not always possible to reliably identify which parts of the image have influenced the model and in what manner. Thus, existing approaches do not permit modelling of heart age using interpretable measures of cardiovascular structure and function.

CMR radiomics analysis allows extraction of a large number of highly detailed measures of cardiac shape and myocardial tissue character^7^, which provide new information over conventional metrics. It may be possible to develop an interpretable model of biological heart age using CMR radiomics cardiovascular phenotypes. However, this has not been previously reported.

In the present study, we used CMR radiomics features to develop a heart age estimation model in 29,996 healthy men and women from the UK Biobank. We examined associations of radiomics features with heart age in men and women, observing sex-differential patterns. We subtracted actual age from model estimated heart age to calculate a “heart age delta”, which we considered as a measure of accelerated (or decelerated) heart aging. We performed a phenome-wide association study (PheWAS) of 701 exposures with heart age delta.

## Methods

### Setting and participants

The UK Biobank is a cohort study including over 500,000 participants. Individuals aged 40–69 years old were identified from National Health Service (NHS) registers and recruited between 2006 and 2010. Baseline assessment comprised characterisation of participant demographics, lifestyle, and medical history, a series of physical measures, and blood sampling. Participants who were unable to consent or complete baseline assessment due to discomfort or ill health were not recruited. The UK Biobank protocol is publicly available^8^. The UK Biobank Imaging Study, which includes CMR, launched in 2015 and is currently underway with the aim of scanning a random 100,000 of the original participants.

### Study sample

**Figure 1:**
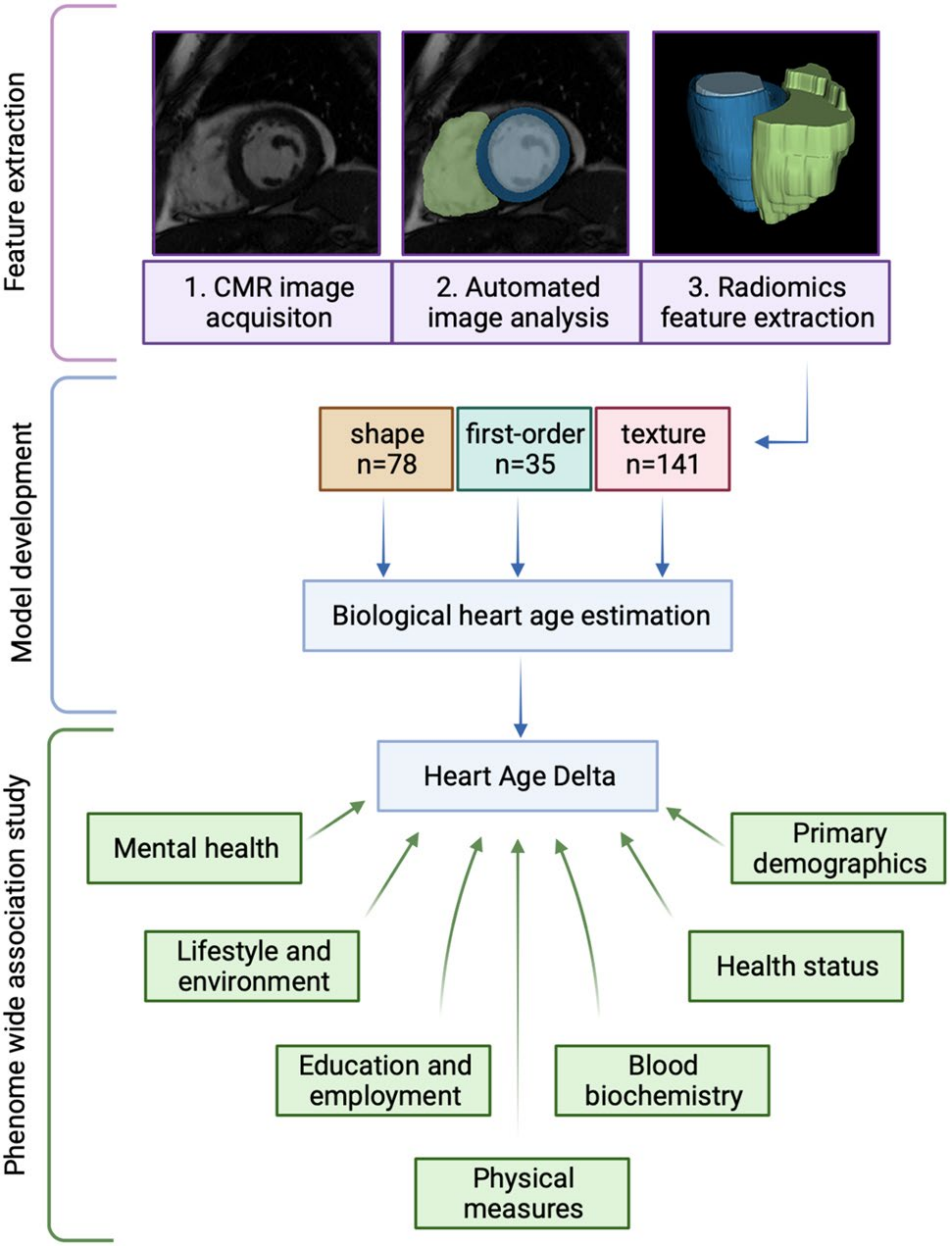
Summary of study workflow.

We included all UK Biobank participants with CMR data available and without cardiovascular disease, as ascertained from baseline assessment and linked Hospital Episode Statistic records (Supplementary Table 1). We limited to individuals from White ethnic backgrounds, to remove noise from ethnicity-related variation of CMR phenotypes. There was inadequate sample size to build separate models for other ethnicities. A complete overview of the study methods including sample selection and all subsequent analyses is presented in Fig. 1 and Supplementary Fig. 1 in accordance with TRIPOD recommendations.

### CMR image acquisition

CMR imaging in the UK Biobank is performed using standardised equipment and staff training^9^. The acquisition protocol is detailed elsewhere^10^. CMR scans were performed using 1.5 Tesla scanners (MAGNETOM Aera, Syngo Platform VD13A, Siemens Healthcare, Erlangen, Germany). Cardiac function was assessed with three long axis cines (horizontal long axis, vertical long axis, left ventricular outflow tract) and a complete short axis stack covering the left ventricle (LV) and right ventricle (RV) acquired at one slice per breath hold using balanced steady-state free precession sequences. Typical acquisition parameters were as follows: Repetition Time/Echo Time = 2.6.1.1 ms, flip angle 80°, Grappa factor 2, voxel size 1.8 mm × 1.8 mm × 8.0 mm (6.0 mm for long axis). The actual temporal resolution of 32 ms was interpolated to 50 phases per cardiac cycle (~ 20 ms). Aside from distortion correction, no signal or image filtering was applied.

### CMR image segmentation

The first 5000 UK Biobank CMR scans were manually segmented using CVI42 post-processing software (Version 5.1.1, Circle Cardiovascular Imaging Inc., Calgary, Canada). The analysis protocol has been previously published^11^. In brief, LV endocardial and epicardial borders were contoured in end-diastole and end-systole in the short axis stack images. End-diastole was defined as the first phase of the acquisition. End-systole was selected as the cardiac phase at which the mid-ventricular LV intra-cavity blood pool appeared smallest by visual inspection. The LV papillary muscles were considered part of the blood pool (excluded from LV mass). The right ventricular (RV) endocardial borders were segmented in end-diastole and end-systole. The most basal slice for the LV was included in the segmentation if at least half of the LV blood pool circumference was surrounded by myocardium. The pulmonary valve plane was used to define the most basal RV slice, with volumes below the valve plane considered as part of the RV. This ground truth manual analysis set was used to develop a fully automated image analysis pipeline with inbuilt quality control, which is described elsewhere^12^. Details of reproducibility performance of the automated algorithm are available in dedicated publications^12,13^. The segmentations generated from this automated pipeline were used to define three regions of interest (ROIs) for radiomics analysis: LV, RV, and LV myocardium.

### CMR radiomics feature extraction

CMR radiomics is a novel image analysis method that allows deeper phenotyping of cardiac structure and myocardial tissue character^7^. On one hand, compared to conventional cardiac indices such as cardiac volumes and ejection fraction, radiomics encode a highly rich set of advanced shape, size, intensity and textural characteristics, including sphericity, compactness, eccentricity, elongation, average intensity, entropy, texture uniformity, texture coarseness, localised contrast, and structural continuity. On the other hand, compared to black box deep learning based approaches, radiomics features permit development of cardiovascular statistical or machine learning models that are both predictive and interpretable. Prior to feature extraction, we applied image normalisation by means of histogram matching using as reference one of the available studies to reduce intensity variations related to the acquisition process. We extracted radiomics shape features from all three ROIs and signal intensity based features (first-order, texture) from the LV myocardium. We included all radiomics features available from the Pyradiomics open source platform version 2.2.0^14^, except features with poor repeatability, identified from our previous work^15^. A total of 254 radiomics features per study were included, comprising 78 shape, 35 first order, and 141 texture features. The full list of radiomics features included in modelling is presented in Supplementary Table 2. Further background to CMR radiomics is available in a dedicated review paper^7^.

### Model building

Analysis was performed using scikit-learn in Python^16^. We trained models separately in men and women. The radiomics features were set as the independent variables (predictors, model inputs) and the age at imaging as the dependent variable (model output). We implemented a Bayesian ridge regression model with tenfold cross-validation. This method was selected due to its reported ability to handle multicollinearity, which we expect between radiomics features^17,18^. Furthermore, this model does not require splitting of the data into training and test sets for hyperparametric tuning as it does not have many parameters to run. Instead, we may apply k-fold cross-validation and then include all the subjects in the model for further analysis, as we do in our study. The radiomics features were set as the independent variables (predictors, model inputs) and the age at imaging as the dependent variable (model output). We adjusted radiomics features for body size variation using height and weight measured at imaging. These confounds were regressed from the radiomics features using linear regression model where each feature was dependent variable and the confounds were the independent variables. The de-confounded radiomics features were subsequently standardized to have zero-mean and unit-variance before fitting them to the model. Then we trained and validated the model using tenfold cross-validation, i.e., the samples were divided into 10 folds: 9 folds to train the model and one fold for validation. To evaluate the model performance, for each fold, we calculated mean absolute error (MAE), coefficient of determination (R^2^), and correlation between predicted heart age and actual age.

We calculated the difference between the model predicted heart age and the actual age, to derive a heart age delta variable for all participants. Heart age delta is, in other words, the residuals from the model and quantifies the degree of variation of actual age from the predicted heart age. As such, a positive heart age delta indicates that the individuals’ heart age is older than their actual age, whilst a negative heart age delta indicates that their heart age in younger than their actual age.

As previously described in brain age modelling, we found that heart age was systematically underestimated for older subjects and overestimated for younger subjects, whilst providing the most accurate estimates for subjects with ages closer to the sample mean. This phenomenon is known as regression dilution bias and is reported in a range of settings^19^, including brain age estimation^20,21^. Within brain age estimation correction methods have been proposed. We sought to describe participants’ heart age without dependency on their current age. As such, we adopted a statistical bias-adjustment method, to correct estimated heart age, as used previously to correct brain age^22^. First, we calculated the regression line between heart age delta and the actual age in the training sets as shown in Eq. (1):

1 $$D = \alpha *\Omega +\beta$$

where ${D}$ is the heart age delta in the training data, ${\alpha }$ and $\beta$ are the slope and the intercept, respectively, of the linear regression model and $\Omega$ represents the actual age.

Then we used the estimated parameters to correct the predicted heart age in the validation data as shown in Eq. (2):

2 $$CPHA = Predicted \; heart \; age - (\alpha *\Omega +\beta )$$

where $\mathrm{CPHA}$ stands for corrected predicted heart age (bias free).

To verify the impact of the bias correction method, we calculated the correlation between predicted heart age and actual age, and the correlation between actual age and heart age delta before and after correction.

We used heart age delta, calculated from the final bias-corrected model, to investigate the association of a wide range of exposures with heart aging.

### Phenotypic alterations and heart age

Statistical analysis was performed using scikit-learn and seaborn in Python^16,23^. We calculated the correlation of each radiomics feature with biological heart age. Thus, we were able to quantitively characterise phenotypic changes in the hearts of men and women with increasing biological heart age. The radiomics features were de-confounded (height, weight, age) before performing correlation. We report Pearson correlation coefficient (r) and Bonferroni corrected p-values (corrected p-value = p-value * number of tests) at $\alpha =0.05$, which were converted to − log10 in the manuscript figures for better visualization. We organised the results into radiomics feature category (shape, first-order, texture) and sorted by “most informative” features first, designated based on the strength and statistical significance of the correlation.

### PheWAS

To investigate the relative importance of exposures associated with heart aging, we calculated correlations of a wide range of exposures with heart age delta and examined the magnitude and direction of these relationships. We reviewed all exposure variables recorded in the UK Biobank. From these, we selected 701 variables for inclusion in the study. These included 666 exposures for both men and women, plus 30 female-specific and 5 male-specific factors. We grouped these into the following categories: (1) Abdominal MRI; (2) Blood biomarkers; (3) Cognitive function; (4) Education and employment; (5) Early life factors; (6) Health related outcomes; (7) Lifestyle and environment; (8) Mental health; (9) Physical measures; (10) Primary demographics; (11) Self-reported health conditions; (12) Female specific factors; (13) Male specific factors.

We first regressed confounds (age, height, weight) from the exposure variables using linear regression. Then we calculated the correlation between heart age delta and the de-confounded exposures. A positive correlation indicates that increasing levels of the exposure are linked to larger heart age delta, suggesting older heart age compared to actual age. Whilst a negative correlation indicates the reverse. We present Pearson correlation coefficients (r) and Bonferroni corrected p-values at $\alpha =0.05$ (association is significant if the corrected p-value (p-value * number of tests) < 0.05). The number of tests equal to the number of exposures included in each of the 13 categories mentioned above. For example, the number of tests in abdominal MRI is 16. Then, each p-value within this group is multiplied by 16 and the resulting (corrected) p-value is considered significant if < 0.05. The corrected p-values were converted to − log10 for better visualization in the figures. The full list of exposure variables and granular results are available in Supplementary Files 1 and 2.

### Ethics statement

This study complies with the Declaration of Helsinki; the work was covered by the ethical approval for UK Biobank studies from the National Health Service (NHS) National Research Ethics Service on 17th June 2011 (Ref 11/NW/0382) and extended on 18 June 2021 (Ref 21/NW/0157) with written informed consent obtained from all participants.

## Results

### Baseline characteristics

CMR segmentations were available for 32,121 participants. We excluded 1185 individuals with cardiovascular disease and 940 participants from ethnic backgrounds other than White. Thus, 15,920 women and 14,076 men were included in the analysis. The age range at time of imaging was 45–82 years old for both men and women with similar age distribution for both sexes (Table 1).

**Table 1:** Baseline participant characteristics.

	Women	Men
Number of participants	15,920	14,076
Age (years)	Mean 62.7 (± 7.3) Median 63 [57, 68]	Mean 63.8 (± 7.6) Median 65 [58, 70]
Townsend deprivation score	Mean − 1.9 (± 2.6) Median − 2.6 [− 3.8, − 0.6]	Mean − 2 (± 2.6) Median − 2.7 [− 3.9, − 0.7]
Height (m)	Mean 163.7 (± 6.3) Median 164 [160, 168]	Mean 177.4 (± 6.6) Median 177 [173, 182]
Weight (kg)	Mean 68 (± 12.7) Median 66 [60, 75]	Mean 83.1 (± 13.2) Median 81 [74, 90]
BMI (kg/m^2^)	Mean 26 (± 4.5) Median 25.1 [22.9, 28.3]	Mean 27 (± 3.8) Median 26.6 [24.5, 29]
Systolic blood pressure (mmHg)	Mean 133.4 (± 19.1) Median 131 [120, 145]	Mean 140.9 (± 17.3) Median 139 [129, 151]
Diastolic blood pressure (mmHg)	Mean 79.3 (± 10.2) Median 79 [72, 86]	Mean 83.7 (± 10.1) Median 83 [77, 90]
Multimorbidity (number of non-cancer illnesses)	Mean 2.8 (± 3.3) Median 2.0 [1.0, 4.0]	Mean 2.5 (± 2.8) Median 2.0 [1.0, 3.0]
Current smoker	826 (5%) smokers	1029 (7.3%)
Cholesterol (mm/L)	Mean 5.9 (± 1.1) Median 5.8 [5.1, 6.5]	Mean 5.6 (± 1.1) Median 5.6 [4.9, 6.3]
Glycosylated haemoglobin (mmol/mol)	Mean 34.7 (± 4.5) Median 34.4 [32.2, 36.8]	Mean 35 (± 5.3) Median 34.6 [32.2, 37.0]

Categorical variables are as number (percentage). Continuous variables are reported as mean (± standard deviation) and median [25th percentile, 75th percentile]. All measures are as recorded at the imaging visit, except for serum cholesterol and glycosylated haemoglobin, which are from baseline.

### Model performance

As expected, there was bias in the uncorrected heart age estimation model demonstrated by the correlation between heart age delta and the actual age (Supplementary Figs. 2 and 3, Panel A). The proposed bias-free correction method was successful in reducing this correlation to near zero, indicating removal of the age dependency, and hence related bias, from the model (Fig. 3, Panel C). Model performance was poorer in men compared to women, with a larger MAE (5.48 vs 4.95) and lower R^2^ value (0.22 vs 0.31).

**Table 2:** Data characteristics and model performance.

Matrices	Women	Men
Number of participants	15,920	14,076
Age (years)	62.7 (± 7.3)	63.8 (± 7.6)
Age range	45–82	45–82
Number of features	254	254
Mean absolute error	4.95	5.48
R squared	0.31	0.22
Correlation of chronological age with predicted age	0.90	0.91
Correlation of heart age delta with actual age	− 0.01	− 0.01

### Correlation of radiomics features with heart age

**Figure 2:**
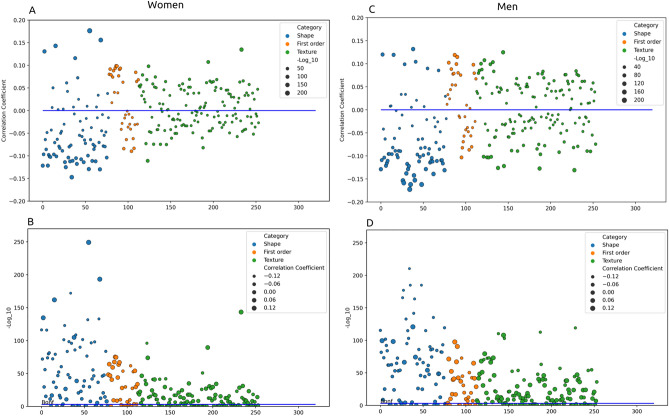
Association of radiomics features with heart age. Each circle represents an individual radiomics feature. The blue, orange, and green circles indicate individual radiomics shape, first order, and texture features respectively. Correlation coefficients are from Pearson correlation of radiomics features against heart age. The p-values are corrected for multiple testing and converted to − log10, so larger values indicated smaller (more significant, p-value * 254 < 0.05) values. (**A** and **B**) are the results for women. (**C** and **D**) are the results for men. (**A** and **C**) The distance of each circle from the blue line indicates the magnitude of the correlation coefficient (as per y-axis) with heart age. The size of the circles reflects magnitude of the p-value with larger circles indicating smaller p-values. (**B** and **D**) The distance from the blue line indicates size of the p-value (level of significance) and the size of the circle indicates magnitude of the correlation coefficient. Horizontal line depicts the Bonferroni threshold of significance (p-value * 254 < 0.05) for multiple comparisons (a = 0.05).

In men, 76% (193/254) of the radiomics features associated with biological heart age, compared to 70% (177/254) in women (Fig. 2, Supplementary Table 2).

For both men and women, the most informative features were from the shape category. There were significant (p-value * 254 < 0.05) associations of heart age with 85% and 83% of the radiomics shape features in men and women, respectively. In men, the most informative (most significant (p-value * 254 < 0.05) and greatest magnitude) associations appeared with RV shape features; advancing heart age was associated with smaller RV axis dimensions (in all directions), smaller RV volumes, and smaller RV internal cavity surface area (Supplementary Table 2). Amongst women, the most informative associations were with shape features extracted from the LV myocardium and the LV cavity. These included greater sphericity of the LV myocardium, and greater surface area to volume ratio of the LV cavity and of the LV myocardium with increasing heart age. There were also associations between greater heart age and smaller LV and RV cavity sizes in women, but these appeared to be less prominent features of aging than for men (Supplementary Table 2).

**Figure 3:**
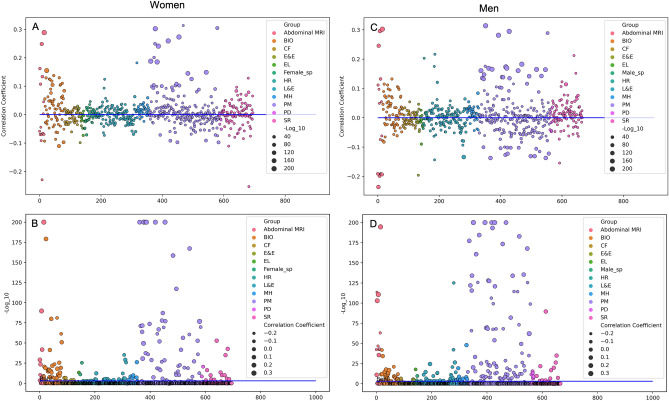
Results from the phenome wide association study. Associations of exposures recorded in UK Biobank with heart age delta expressed using Pearson correlation coefficient with corrected p-value (− log10). Each circle represents an individual exposure. In (**A** and **C**), correlation coefficient is shown on the y-axis and the magnitude of p-value is represented by the size of the circles (larger circles indicate more significant (p-value * number of tests < 0.05) results). (**B** and **D**) have − log10 corrected p-value on the y-axis, thus distance from the line represents significance level and size of the circles represents magnitude of the correlation. Bio: biochemistry; CF: cognitive function; E&E: education and employment; EL: early life factors; Female_sp: female specific factors; Male_sp: male specific factors; HR: health related outcomes; MRI: magnetic resonance imaging; L&E: lifestyle and environment; MH: mental health; PM: physical measures; PD: primary demographics; SR: self-reported health conditions.

The significant associations (p-value * 254 < 0.05) between heart age and signal intensity based radiomics features (first order, texture) extracted from the LV myocardium appeared notably more numerous and of greater magnitude in men compared to women (72% vs 64%, Fig. 3). With increasing heart age, there was observation of a brighter myocardium and greater variation in LV myocardial signal intensities. Overall, the direction of change in these features appeared similar but less pronounced in women compared to men (Supplementary Table 2). The most defining radiomics features of biological heart age are summarised in Supplementary Fig. 4.

### PheWAS

Granular results for the PheWAS are presented in Supplementary File 1 (men) and Supplementary File 2 (women) and visualised in Fig. 3. We additionally summarise all significant (corrected p-value < 0.05) associations in Supplementary Table 3. There was a greater number of statistically significant (corrected p-value < 0.05) exposure associations with heart age delta in men compared to women (27.1% vs 20.2%), and in general, the magnitude of associations appeared larger for men (Fig. 3). There was overlap of 16.2% associations for men and women (Supplementary Table 4).

### Obesity

**Figure 4:**
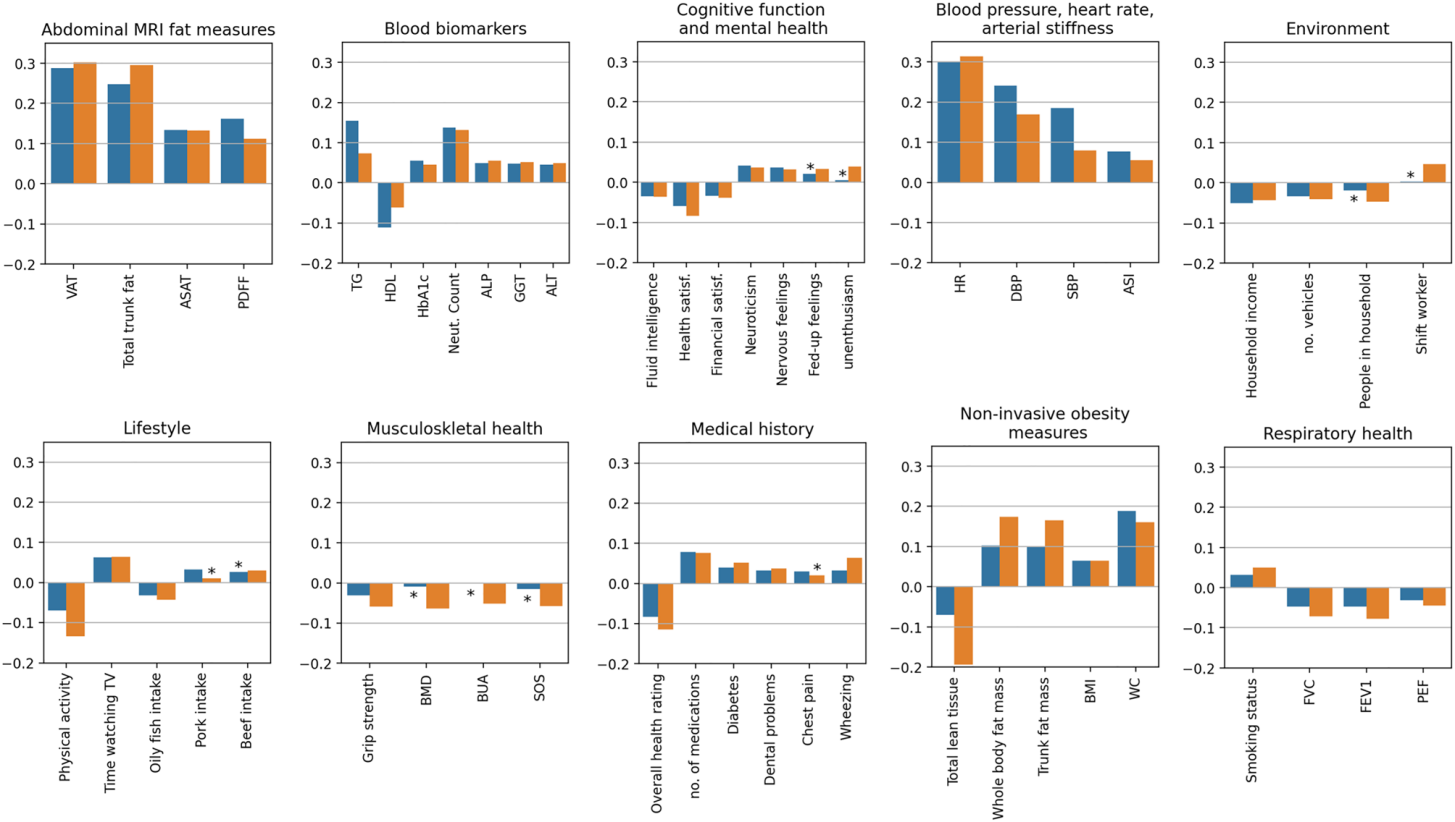
Selected results from the PheWAS. Results are Pearson correlation coefficients of exposures with heart age delta in men (orange) and women (blue). Positive correlations indicate exposures linked to greater heart age delta (accelerated heart aging) and negative correlations indicate exposures linked to smaller heart age delta (decelerated heart aging). Asterix indicates results that are not statistically significant (p-value * number of tests > 0.05). ALP: alkaline phosphatase; ALT: alanine aminotransferase; ASAT: abdominal subcutaneous adipose tissue volume; ASI: arterial stiffness index; BMD: bone mineral density; BMI: body mass index; BUA: bone ultrasound attenuation; DBP: diastolic blood pressure; FEV1: forced expiratory volume in 1 s; FVC: forced vital capacity; GGT: gamma glutamyl transferase; HbA1c: serum glycosylated haemoglobin; HDL: high density lipoprotein; HR: heart rate; LDL: low density lipoprotein; Neut: neutrophil; PDFF: Proton density fat fraction; PEF: peak expiratory flow; SBP: systolic blood pressure; TG: triglyceride level; TV: television; VAT: visceral adipose tissue volume; WC: waist circumference. Please note, for “health satisf.” “financial satisf.”, and “overall health rating” variables, the UK Biobank standard coding tables allocate higher score to poorer ratings, here we reverse the coding for more intuitive interpretation.

The most convincing positive correlations were between heart age delta and different measures of obesity (Figs. 3, 4). The strongest correlations, for both men and women, were observed with abdominal magnetic resonance imaging (MRI) measures of visceral adipose tissue volume (VAT), total trunk fat volume, abdominal subcutaneous adipose tissue volume, and total adipose tissue volume. Notably, higher Proton Density Fat Fraction (PDFF), an MRI measure of liver fat, was also positively and significantly (p-value = 2.6 × 10^–13^ for women, p-value = 1.9 × 10^–5^ for men) correlated with higher heart age delta (accelerated heart aging). Consistently, greater lean tissue volume (measured from various body locations) correlated with significantly (p-value 0.0001 for women, p-value = 4.3 × 10^–28^ for men) smaller heart age delta for both men and women.

Body composition measures were also obtained using a non-invasive analysis method, which works by transmitting a low-level current through the body and analysing the ‘impedance’ or current lost in flow. As with the abdominal MRI metrics, we found that these impedance measures of obesity (e.g., whole body fat mass) were linked to greater heart aging, whilst impedance measures of lean/muscle mass (e.g., whole body fat-free mass) were linked to smaller heart age deltas.

More established anthropometric measures of obesity, e.g., body mass index (BMI) and waist circumference, also associated positively with heart age delta. The correlations with BMI appeared weaker than the MRI or impedance obesity measures. For example, in men, heart age delta was positively correlated with abdominal VAT (cc = 0.30; p-value = 2.93 × 10^–85^), impedance trunk fat mass (r = 0.17; p-value = 7.83 × 10^–81^), and BMI (r = 0.06; p-value = 1.27 × 10^–11^). Interestingly, higher self-reported comparative body size in childhood was linked to decelerated heart aging for both women (r = − 0.07; p-value = 1.68 × 10^–6^) and men (r = − 0.09; p-value = 2.10 × 10^–8^).

### Blood biomarkers

In line with the physical and imaging measures of obesity, adverse serum lipid profile also appeared strongly associated with greater heart aging in both men and women (Fig. 3, Supplementary Table 3). Greater heart age delta was linked to higher triglyceride, higher low density lipoprotein (LDL) cholesterol, and lower high density lipoprotein (HDL) cholesterol levels. Overall, the pattern of associations with adverse serum lipid markers appeared more consistent for women, with generally stronger associations and significant (corrected p-value < 0.05) correlations across a larger number of metrics than men. For example, of all the lipid metrics, triglyceride level had the strongest correlations with heart age delta for both men and women, however, the correlation was stronger in women (r = 0.15, p-value = 1.57 × 10^–78^) than in men (r = 0.07; p-value = 1.42 × 10^–15^).

In men, serum liver metrics (e.g., alkaline phosphatase, gamma glutamyl transferase, alanine aminotransferase) also appeared as significant (p-value = 2.2 × 10^–8^, 3.3 × 10^–7^,1.9 × 10^–6^ respectively) positive correlates of heart age delta. These correlations were also seen for women but were of slightly smaller magnitude. Interestingly, the liver fat associations (as per the MRI PDFF metric) appeared stronger in women than in men. For both men and women, poorer glycaemic control, measured by higher glycosylated haemoglobin (HbA1c) was linked to larger heart age delta. We also observed links between blood markers of systematic inflammation (e.g., neutrophil count, C reactive protein) and greater heart age delta.

### Blood pressure, heart rate, and arterial stiffness

Faster heart rate appeared as a consistent and strong positive correlate of heart age delta for men (r = 0.31; p-value = 1.85 × 10^–273^) and women (r = 0.30; p-value = 5.80 × 10^–284^). Higher systolic and diastolic blood pressure measurements (SBP, DBP) appeared as positive correlates of heart age delta; these associations appeared more convincing in women than in men. In women correlation of heart age delta with both SBP (r = 0.19; p-value = 2.64 × 10^–96^) and DBP (r = 0.24; p = 6.68 × 10^–165^) was stronger and more consistent than for men (DBP: r = 0.17; p = 1.52 × 10^–70^; SBP: r = 0.08; p = 4.00 × 10^–15^). Greater pulse wave arterial stiffness index positively correlated with heart age delta in women (r = 0.08; p-value = 6.29 × 10^–17^) and in men (r = 0.05; p-value = 2.26 × 10^–7^).

### Respiratory function

Better lung function assessed using spirometry correlated with decelerated heart aging. For example, higher forced vital capacity, peak expiratory flow, and forced expiratory volume were linked to lower heart age delta in both men and women, but with slightly stronger associations and a little more consistency in men (Fig. 3, Supplementary Table 4).

### Musculoskeletal health

Higher hand grip strength was negatively correlated to heart age delta in both men (r = − 0.06; p-value = 1.53 × 10^–9^) and women (r = − 0.03; p-value = 0.04). In men, measures of better bone health from quantitative heel ultrasound (speed of sound, broadband ultrasound attenuation, estimated bone mineral density) were all linked to smaller heart age delta. These associations with bone health were not observed in women.

### Cognitive function

Better performance on cognitive function tests was linked to less heart aging in both men and women. For example, higher fluid intelligence score correlated with lower heart age delta in women (r = − 0.03; p-value = 1.20 × 10^–3^) and men (r = − 0.04; p-value = 1.65 × 10^–3^).

### Lifestyle and environment

For both men and women, measures representing greater levels of socio-economic deprivation were linked to greater heart aging. Greater household income and greater number of vehicles in the household were linked to smaller heart age delta. In men, having a job involving shift work was linked to higher heart age delta.

Higher physical activity levels were linked to lower heart age delta for both men and women, but with slightly greater strength of correlation for men. For both, longer time watching television was linked to larger heart age delta. Smoking was linked to larger heart age delta for both men and women. In women greater pork intake and in men greater beef intake, correlated with higher heart age deltas. In both men and women, greater oily fish intake and greater intake of cereals were linked to smaller heart age delta.

### Mental health

Higher health satisfaction and financial satisfaction scores were significant (p-value = 1.6 × 10^–21^, 0.0002 for men & 4.7 × 10^–12^, 0.0008 for women respectively) negative correlates of heart age delta for both men and women. For both sexes, higher neuroticism score, and greater tendency to “nervous” feelings, or “worried/anxious” feelings were all correlated with significantly (p-value = 0.001, 0.03 for men and 5.3 × 10^–5^, 0.0003 respectively) greater heart age delta. In men, there were additional significant (p-value = 0.01) associations with variables indicating low mood or depression, which did not appear significantly (p-value = 1.00) correlated with heart age delta in women. For example, in men, lower happiness score, greater “miserableness”, low enthusiasm, and greater tendency to “fed-up” feelings all appeared as significant (p-value = 0.003, 0.01, 0.0001, 0.03 respectively) positive correlates of heart age delta.

### Medical history

In both men and women, poorer self-reported health rating, greater number of medications taken, and history of chronic disease or disability were all linked to greater heart aging. Self-reported symptoms of chest pain or wheezing correlated with higher heart age delta, as did a history of dental problems. History of medication use for cholesterol, diabetes, or hypertension as well as a clinical diagnosis of diabetes associated positively to heart age delta.

### Sex-specific factors

In women, older age at both time of the first live birth and last live birth was associated with lower heart age delta. In men, “number of children fathered” was linked to lower heart age delta. In men, there was a small but statistically significant (p-value = 0.01) correlation between lower heart age delta and a more extensive balding pattern (Type 4 pattern, as per Giles et al.^24^).

## Discussion

### Summary of findings

In this large population-based cohort free from cardiovascular disease, we developed a novel heart age estimation tool using CMR radiomics measures of cardiac shape and myocardial character. There was evidence of differential heart aging in men and women. Men had extensive age-related phenotypic alterations across all radiomics feature categories (shape, first-order, texture), suggesting that in men, older heart age is linked to both gross morphological alterations of the heart and important alterations in the LV myocardium. Whilst women also showed some alterations of LV myocardium texture features, these were less extensive than in men. In men, changes of RV shape appeared to be the most important feature of heart aging, whereas in women geometric alterations of the LV and myocardium appeared more prominent.

In the PheWAS we demonstrate the feasibility and validity of using heart age delta, derived from our heart age estimation model, as a measure of the rate of cardiac aging. The strongest correlates of accelerated heart aging were measures of obesity, adverse serum lipid markers, hypertension, diabetes, heart rate, income, multimorbidity, musculoskeletal health, and respiratory health. The presented heart age model may now be used to investigate determinants of heart aging in dedicated hypothesis driven studies.

### Comparison with existing work

Our observations of smaller LV and RV sizes with older heart age are consistent with previous reports of conventional heart metrics in healthy aging^5^. The dominance of RV morphological alterations in men and LV and myocardial geometric alterations in women has not been previously reported. Furthermore, the novel radiomics features allowed appreciation of more detailed phenotypic alterations, for example alongside reduction in RV cavity axis dimensions and volumes we also observed reduction of the RV cavity internal surface area, which may indicate attenuation of RV endocardial trabeculations with aging. Additionally, we found greater sphericity of the overall LV shape, reduced height of the LV (and accordingly, greater surface area to volume ratio of the LV myocardium) to be dominant features of heart aging in women.

We observed convincing evidence of alterations at the myocardial level with increasing heart age in men, but less so in women. Ours is the first study to report these myocardial texture patterns using CMR radiomics features. However, our results are consistent with previous studies using conventional measures of myocardial character, such as, myocardial native T1, which report clear age trends in men but inconsistent results in women^25–27^.

We found significant correlations between spirometry measures indicating better lung function and smaller heart delta. These correlations, along with the adverse heart age effect of smoking, appeared more consistent in men than women. Given the strong dependency of RV size and function on respiratory health, these more convincing cardio-respiratory relationships in men may explain the dominance of RV alterations seen in aging of male hearts. The interconnected relationship between cardiovascular and respiratory health is of course well described^28^ and previous epidemiologic research has suggested links between poorer spirometry metrics and greater risk of cardiovascular mortality^29^.

The strongest correlates of greater heart aging in our study were measures of obesity. Interestingly, measures of visceral and central obesity appeared to correlate more strongly with accelerated heart aging than measures of subcutaneous obesity. The links between obesity and adverse conventional LV CMR measures have been previously reported using anthropometric measures of obesity^30^ and measures of visceral adiposity^31^. However, the key importance of obesity in heart aging in the general population has not been highlighted previously.

Furthermore, we found significant correlations of greater heart aging with higher liver fat and adverse serum liver markers. Indeed, there is growing interest in the importance of the heart-liver axis and recent reports have emphasised the links between non-alcoholic fatty liver disease and poorer cardiovascular health^32^. Our findings add strength to these reports and demonstrate novel links between liver adiposity and heart aging in a population cohort without cardiovascular disease, which merits further dedicated investigation.

We demonstrate multiple further cross-system interactions with heart aging, including with brain and musculoskeletal health, which are broadly consistent with previous reports of multi-system health^33,34^. Indeed, measures of multimorbidity also appeared as important correlates of heart aging. There is growing support for consideration of disease patterns within multisystem contexts^35–37^. Our findings in association with heart aging support such approaches.

An interesting observation was the strong correlation of faster heart rate with heart aging. Previous work has demonstrated faster resting heart rate as a reliable predictor of cardiovascular outcomes in men and women^38^. However, the underlying mechanisms for these relationships are not fully understood. Our results suggest that faster heart rates are linked to aging related alterations of the cardiac phenotype. Thus, our findings support heart rate as an indicator of cardiovascular risk and suggest that these effects may be mediated through promotion of adverse cardiovascular remodelling.

We also observed expected correlation of SBP and DBP with heart aging in both men and women, with stronger associations for women. Our observations are in keeping with reports indicating greater end-organ damage in women with hypertension^39,40^ and may explain greater propensity for women to develop heart failure^41^.

Finally, our work also highlights the importance of mental wellbeing and socio-economic factors in heart aging. These relationships are complex and difficult to quantify, however, our results demonstrate consistent correlations across a range of measures. Further study into these important exposures is warranted.

### Strengths and limitations

The large standardised CMR dataset in the UK Biobank and the availability of automated image analysis tools provided the ideal platform to develop the presented heart age estimation model. There is limited ethnic diversity in the UK Biobank (> 98% White ethnicities), as such we were unable to develop models for other ethnicities. As CMR measures are known to vary by ethnicity^5^, our model may not be applicable across different ethnic groups. Furthermore, as we sought to understand heart aging in individuals without overt cardiac disease, the observed relationships in this study may not be applicable in clinical cohorts. In this paper, we demonstrate the initial feasibility of heart age estimation using CMR radiomics data. Wider application of our model requires validation in external independent cohorts and careful scientific scrutiny of reproducibility and validity. As this was the first work to use radiomics feature for heart age estimation, we included all the features available in the present model to avoid excluding any feature that could have a biological meaning and to establish a benchmark for complex methods and feature selection. Application of features selection methods would be appropriate steps in future work. The detailed characterisation of participants permitted examination of associations of a wide range of exposures with heart age delta. The correlations described in the PheWAS are after minimal confounder adjustment. Additionally, the exposures are taken as reported by the UK Biobank; consideration of outlier removal or other sense checks were beyond the scope of this study. In future studies, a more focused hypothesis-driven approach with greater care in preparation of exposures and consideration of more extensive confounder adjustment are needed. Some of the reported exposure associations with heart age delta were statistically significant but very small in magnitude. Whilst these relationships are informative in understanding population-level trends, their value in evaluating individual-level risk is uncertain. Future research is required to evaluate the clinical utility of heart age delta as an indicator of cardiovascular risk and its incremental value over existing approaches.

## Conclusions

We present a novel heart age estimation tool developed using image derived radiomics phenotypes of cardiac shape and myocardial character. We propose heart age delta derived from this model as an indicator of heart aging. We observed a pattern of exposure associations with heart age delta which is consistent with our biological knowledge of cardiovascular health. As such, our findings support the validity of the heart age delta metric to investigate novel determinants of heart aging in population cohorts. Furthermore, this new technique provides a novel method of phenotypic assessment relating to cardiovascular health; whilst this has been shown to be of value in this cross-sectional setting, further studies will be required to assess its predictive value for incident cardiovascular events, whether it generates a risk independent of traditional risk factors, and whether patients treated on the basis of heart age difference respond to treatment as well as those selected on the basis of a specific risk factor.

## Supplementary Information

Supplementary Information 1.

Supplementary Information 2.

Supplementary Information 3.

## Data Availability

This research was conducted using the UK Biobank resource under access application 2964. UK Biobank will make the data available to all bona fide researchers for all types of health-related research that is in the public interest, without preferential or exclusive access for any persons. All researchers will be subject to the same application process and approval criteria as specified by UK Biobank. For more details on the access procedure, see the UK Biobank website: http://www.ukbiobank.ac.uk/register-apply.

## Abbreviations

CPHA: Corrected predicted heart age
CMR: Cardiovascular magnetic resonance
HDL: High density lipoprotein
NHS: National Health Service
LDL: Low density lipoprotein
LV: Left ventricle
MAE: Mean absolute error
MRI: Magnetic resonance imaging
PDFF: Proton density fat fraction
PheWAS: Phenome wide association study
ROI: Regions of interest
RV: Right ventricle
VAT: Visceral adipose tissue volume
