# Supplementary material for: Estimation of biological heart age using cardiovascular magnetic resonance radiomics
Source: Sci Rep. 2022 Jul 27;12:12805. doi: 10.1038/s41598-022-16639-9 (PMC9329281; doi:10.1038/s41598-022-16639-9)
Supplement: Supplementary file 1 — Supplementary Information 1. [file 41598_2022_16639_MOESM1_ESM.pdf]

SUPPLEMENTARY MATERIALS

Estimation of biological heart age using cardiovascular magnetic resonance radiomics

Zahra Raisi-Estabragh (MD, PhD)[1,2*†], Ahmed Salih (MSc)[3,4†], Polyxeni Gkontra (PhD)[4], Angélica Atehortúa (MSc)[4], Petia Radeva (PhD)[4], Ilaria Boscolo Galazzo (PhD)[3], Gloria Menegaz (PhD)[3], Nicholas C. Harvey (MD, PhD)[5,6], Karim Lekadir (PhD)[4], Steffen E. Petersen (MD, DPhil)[1,2,7,8]

1. William Harvey Research Institute, NIHR Barts Biomedical Research Centre, Queen Mary University of London, Charterhouse Square, London, EC1M 6BQ, UK
2. Barts Heart Centre, St Bartholomew's Hospital, Barts Health NHS Trust, West Smithfield, London, EC1A 7BE, UK
3. University of Verona, Department of Computer Science, Verona, 37134, Italy
4. University of Barcelona, Dept. de Matematiques i Informatica, Barcelona, 95P7+JH, Spain
5. MRC Lifecourse Epidemiology Centre, University of Southampton, Southampton, UK
6. NIHR Southampton Biomedical Research Centre, University of Southampton and University Hospital Southampton NHS Foundation Trust, Southampton, UK
7. Health Data Research UK, London, UK
8. Alan Turing Institute, London, UK

†ZRE and AS have contributed equally to this work and share first authorship

Short title: Biological heart age estimation

***Corresponding author:** Dr Zahra Raisi-Estabragh; William Harvey Research Institute, NIHR Barts Biomedical Research Centre, Queen Mary University of London, Charterhouse Square, London, EC1M 6BQ, UK; E-mail: zahraraisi@doctors.org.uk, Telephone: +44 (20) 37658766

Supplementary Figure 1. Overview of the study methods

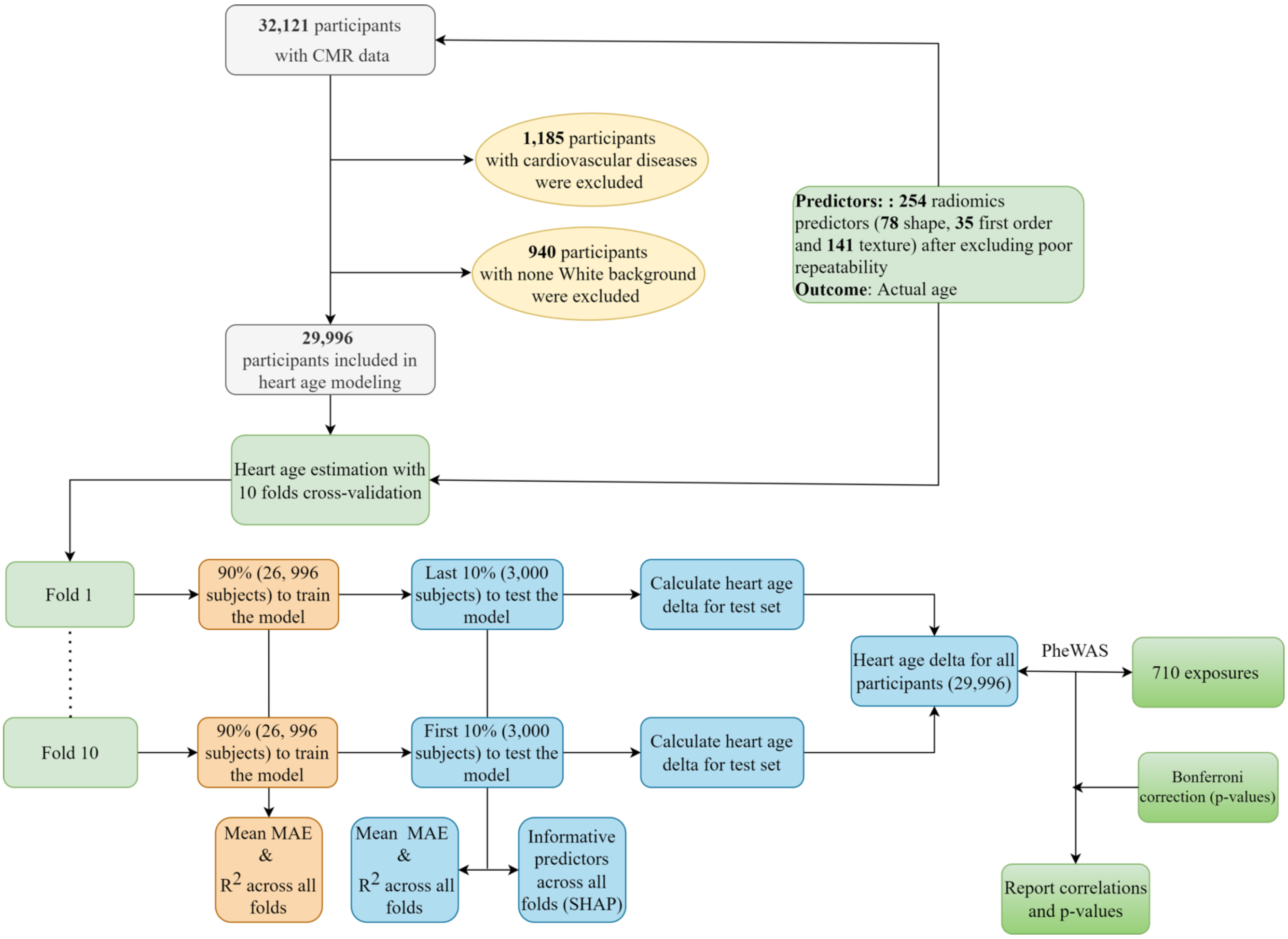

Supplementary Figure 1 footnote. MAE: mean absolute error, SHAP: SHapley Additive exPlanations

Supplementary Figure 2. Correlation of actual age with predicated heart age and heart age delta before and after bias correction (women)

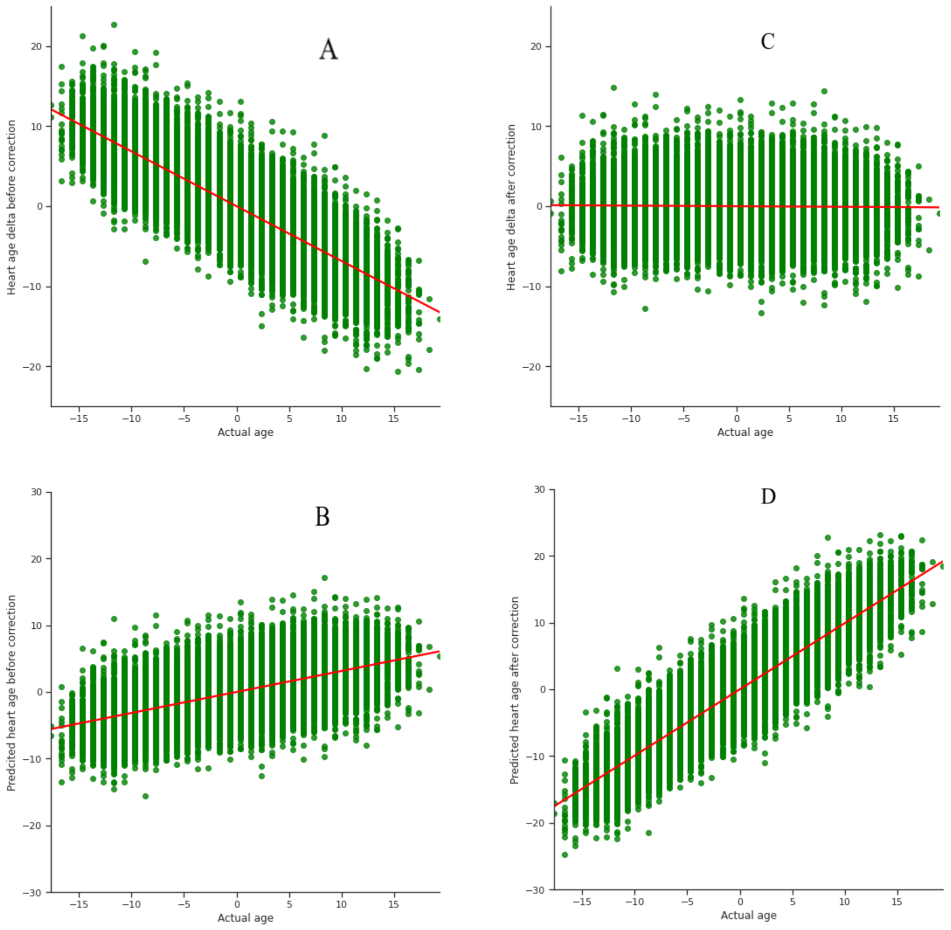

Supplementary Figure 2 footnote. A: correlation of uncorrected heart age delta with actual age. **B:** correlation of corrected heart age delta with actual age. **C:** correlation of uncorrected predicated heart age with actual age. **D:** correlation of corrected predicated heart age with actual age.

Supplementary Figure 3. Correlation of chronological age with predicted heart age and heart age delta before and after bias correction (Men)

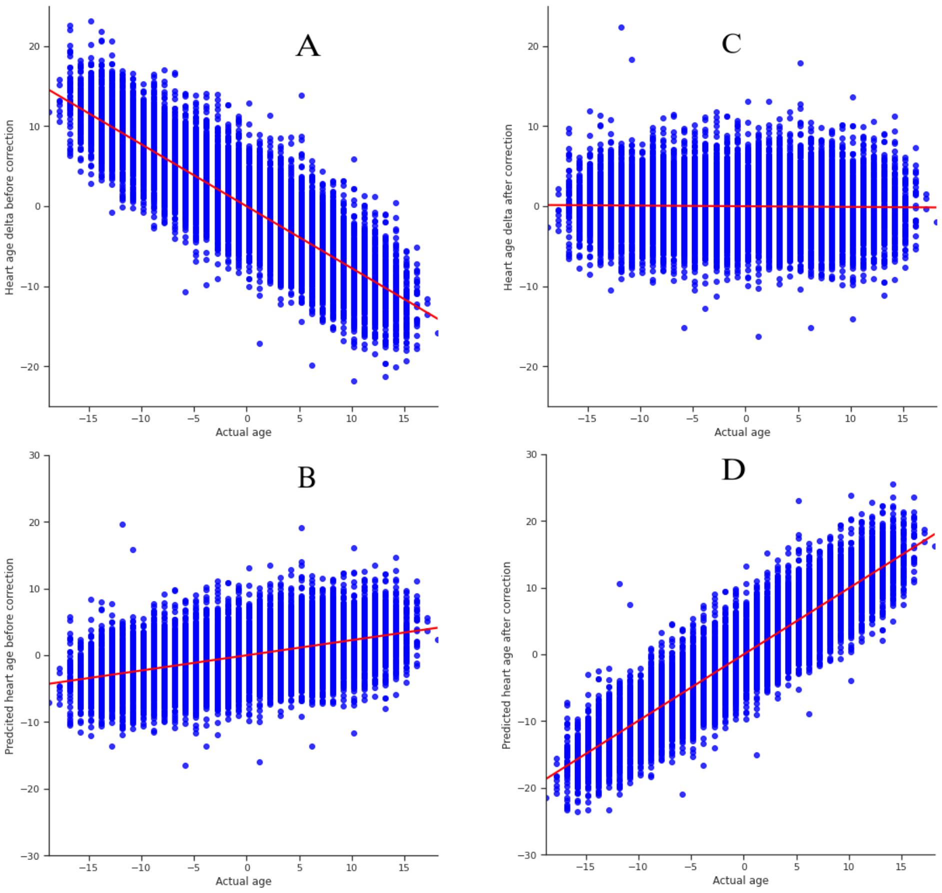

Supplementary Figure 3 footnote. A: correlation of uncorrected heart age delta with actual age. **B:** correlation of corrected heart age delta with actual age. **C:** correlation of uncorrected predicated heart age with actual age. **D:** correlation of corrected predicated heart age with actual age.

Supplementary Figure 4. Top 20 most informative radiomics features associated with heart age for women (A) and men (B)

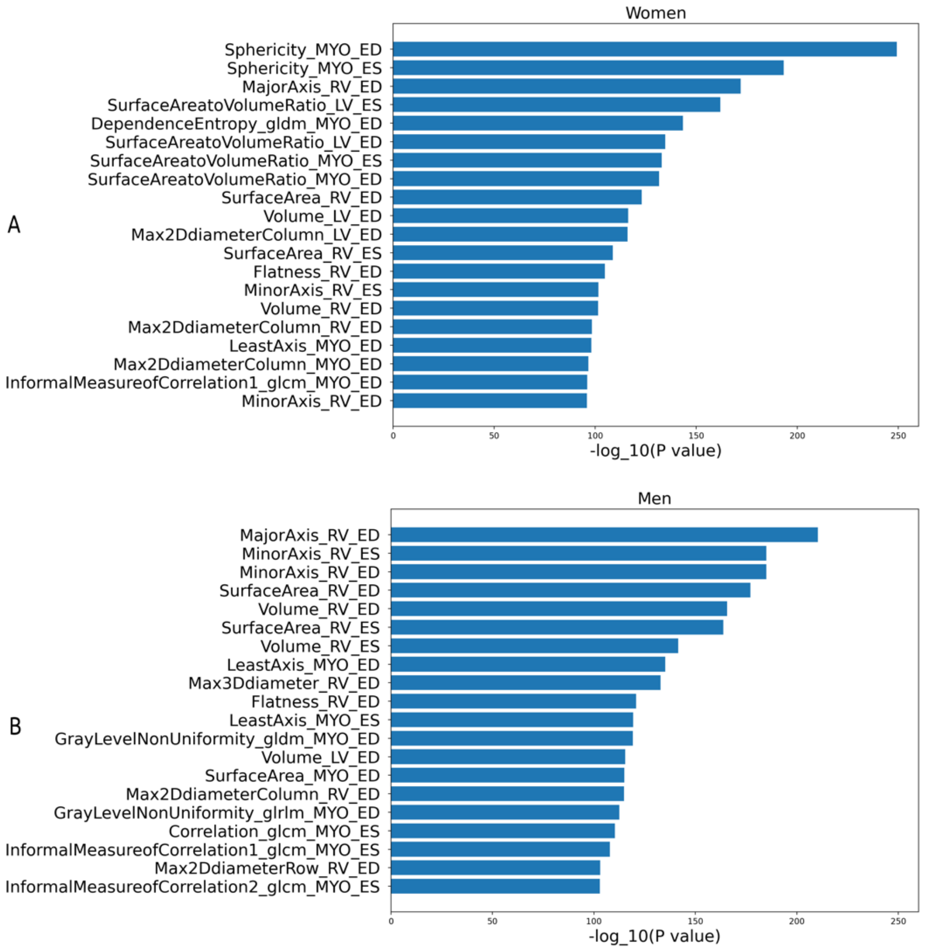

Supplementary Figure 4 footnote. The top 20 most informative feature associations with heart age are shown for women (A) and men (B). The x axis is -log10 p-value. The bars correspond to radiomics features with naming as "Featurename_regionofinterest_cardiacphase"; for texture features subcategory is also indicated. Results are –log10(P-value) for correlation of each feature with heart age. ED: end-diastole; ES: end-systole; GLCM: grey level co-occurrence matrix; GLDM: grey level dependence matrix; GLRLM: grey level run length matrix; LV: left ventricle; MYO: myocardium; RV: right ventricle.

Supplementary Table 1. Approach to definition of cardiovascular disease

Source	Definitions and disease categories
	Ischaemic heart disease
Self-report	Angina (data field 20002)
Self-report	heart attack/myocardial infarction (data field 20002)
Algorithm	AMI on algorithmically defined outcomes (data field 42000)
ICD10	I20 Angina pectoris
ICD10	I21 Acute myocardial infarction
ICD10	I22 Subsequent myocardial infarction
ICD10	I23 Certain current complications following acute myocardial infarction
ICD10	I24 Other acute ischaemic heart diseases
ICD10	I25 Chronic ischaemic heart disease
	Valvular heart disease
Self-report	Mitral stenosis (data field 20002)
Self-report	Mitral regurgitation / incompetence (data field 20002)
Self-report	Aortic valve disease (data field 20002)
Self-Report	Aortic stenosis (data field 20002)
Self-Report	Aortic regurgitation / incompetence (data field 20002)
ICD10	I34.0 Mitral (valve) insufficiency
ICD10	I34.2 Nonrheumatic mitral (valve) stenosis
ICD10	I34.8 Other nonrheumatic mitral valve disorders
ICD10	I34.9 Nonrheumatic mitral valve disorder, unspecified
ICD10	I35.0 Aortic (valve) stenosis
ICD10	I35.1 Aortic (valve) insufficiency
ICD10	I35.2 Aortic (valve) stenosis with insufficiency
ICD10	I35.8 Other aortic valve disorders
ICD10	I35.9 Aortic valve disorder, unspecified
ICD10	I36.0 Nonrheumatic tricuspid (valve) stenosis
ICD10	I36.1 Nonrheumatic tricuspid (valve) insufficiency
ICD10	I36.8 Other nonrheumatic tricuspid valve disorders
ICD10	I36.9 Nonrheumatic tricuspid valve disorder, unspecified
ICD10	I37.0 Pulmonary valve stenosis
ICD10	I37.1 Pulmonary valve insufficiency
ICD10	I37.2 Pulmonary valve stenosis with insufficiency
ICD10	I37.8 Other pulmonary valve disorders
ICD10	I37.9 Pulmonary valve disorder, unspecified
ICD10	I38 Endocarditis, valve unspecified
ICD10	I39.0 Mitral valve disorders in diseases classified elsewhere
ICD10	I39.1 Aortic valve disorders in diseases classified elsewhere
ICD10	I39.3 Pulmonary valve disorders in diseases classified elsewhere
ICD10	I39.4 Multiple valve disorders in diseases classified elsewhere
ICD10	I39.8 Endocarditis, valve unspecified, in diseases classified elsewhere
ICD10	I05 Rheumatic mitral valve diseases
ICD10	I06 Rheumatic aortic valve diseases
ICD10	I07 Rheumatic tricuspid valve diseases
ICD10	I08 Multiple valve diseases
	Non-ischaemic cardiomyopathies
Self-Report	Cardiomyopathy (data field 20002)
Self-Report	Hypertrophic cardiomyopathy (data field 20002)
ICD10	I42.0 Dilated cardiomyopathy
ICD10	I42.1 Obstructive hypertrophic cardiomyopathy
ICD10	I42.2 Other hypertrophic cardiomyopathy
ICD10	I42.5 Other restrictive cardiomyopathy
ICD10	I42.6 Alcoholic cardiomyopathy
ICD10	I42.7 Cardiomyopathy due to drugs and other external agents

ICD10	I42.8 Other cardiomyopathies
ICD10	I42.9 Cardiomyopathy, unspecified
ICD10	I43.0 Cardiomyopathy in infectious and parasitic diseases classified elsewhere
ICD10	I43.1 Cardiomyopathy in metabolic diseases
ICD10	I43.2 Cardiomyopathy in nutritional diseases
ICD10	I43.8 Cardiomyopathy in other diseases classified elsewhere
ICD10	I11.0 Hypertensive heart disease with (congestive) heart failure
ICD10	I11.9 Hypertensive heart disease without (congestive) heart failure
ICD10	I13.0 Hypertensive heart and renal disease with (congestive) heart failure
ICD10	I13.1 Hypertensive heart and renal disease with renal failure
ICD10	I13.2 Hypertensive heart and renal disease with both (congestive) heart failure and renal failure
ICD10	I13.9 Hypertensive heart and renal disease, unspecified
	Heart failure (unspecified aetiology)
Self-Report	Heart failure/pulmonary odema (data field 20002)
ICD10	I50.0 Congestive heart failure
ICD10	I50.1 Left ventricular failure
ICD10	I50.9 Heart failure, unspecified
	Cardiac arrhythmia
Self-Report	Sick sinus syndrome (data field 20002)
Self-Report	SVT / supraventricular tachycardia (data field 20002)
Self-Report	Atrial fibrillation (data field 20002)
Self-Report	Atrial flutter (data field 20002)
ICD10	I44.1 Atrioventricular block, second degree
ICD10	I44.2 Atrioventricular block, complete
ICD10	I45.6 Preexcitation syndrome
ICD10	I46.0 Cardiac arrest with successful resuscitation
ICD10	I46.1 Sudden cardiac death, so described
ICD10	I46.9 Cardiac arrest, unspecified
ICD10	I47.0 Reentry ventricular arrhythmia
ICD10	I47.1 Supraventricular tachycardia
ICD10	I47.2 Ventricular tachycardia
ICD10	I47.9 Paroxysmal tachycardia, unspecified
ICD10	I48.0 Paroxysmal atrial fibrillation
ICD10	I48.1 Persistent atrial fibrillation
ICD10	I48.2 Chronic atrial fibrillation
ICD10	I48.3 Typical atrial flutter
ICD10	I48.4 Atypical atrial flutter
ICD10	I48.9 Atrial fibrillation and atrial flutter, unspecified
ICD10	I49.0 Ventricular fibrillation and flutter
ICD10	I49.5 Sick sinus syndrome

Supplementary Table 1 footnote. Abbreviations: ICD10: international classification of disease 10

Supplementary Table 2. Association of radiomic features with predicted heart age in men and women expressed by Pearson correlation (r) and Bonferroni corrected p-values sorted with the most informative features appearing first

Women				Men			
**Category**	**Feature**	**r**	**p-value**	**Category**	**Feature**	**r**	**p-value**
Shape	Sphericity_MYO_ED	0.18	5.83E-109	Shape	MajorAxis_RV_ED	-0.17	4.39E-92
Shape	Sphericity_MYO_ES	0.16	1.14E-84	Shape	MinorAxis_RV_ES	-0.16	4.98E-81
Shape	MajorAxis_RV_ED	-0.15	1.95E-75	Shape	MinorAxis_RV_ED	-0.16	5.12E-81
Shape	SurfaceAreatoVolumeRatio_LV_ES	0.14	4.89E-71	Shape	SurfaceArea_RV_ED	-0.16	1.29E-77
Texture	DependenceEntropy_gldm_MYO_ED	0.13	5.58E-63	Shape	Volume_RV_ED	-0.15	1.22E-72
Shape	SurfaceAreatoVolumeRatio_LV_ED	0.13	3.44E-59	Shape	SurfaceArea_RV_ES	-0.15	7.60E-72
Shape	SurfaceAreatoVolumeRatio_MYO_ES	-0.13	1.93E-58	Shape	Volume_RV_ES	-0.14	3.23E-62
Shape	SurfaceAreatoVolumeRatio_MYO_ED	-0.13	6.74E-58	Shape	LeastAxis_MYO_ED	-0.14	1.92E-59
Shape	SurfaceArea_RV_ED	-0.13	4.19E-54	Shape	Max3Ddiameter_RV_ED	-0.14	2.14E-58
Shape	Volume_LV_ED	-0.12	3.27E-51	Shape	Flatness_RV_ED	0.13	3.45E-53
Shape	Max2DdiameterColumn_LV_ED	-0.12	4.40E-51	Shape	LeastAxis_MYO_ES	-0.13	1.50E-52
Shape	SurfaceArea_RV_ES	-0.12	6.70E-48	Texture	GrayLevelNonUniformity_gldm_MYO_ED	-0.13	1.83E-52
Shape	Flatness_RV_ED	0.12	3.00E-46	Shape	Volume_LV_ED	-0.13	8.12E-51
Shape	MinorAxis_RV_ES	-0.11	7.85E-45	Shape	SurfaceArea_MYO_ED	-0.13	1.22E-50
Shape	Volume_RV_ED	-0.11	9.88E-45	Shape	Max2DdiameterColumn_RV_ED	-0.13	1.27E-50
Shape	Max2DdiameterColumn_RV_ED	-0.11	1.89E-43	Texture	GrayLevelNonUniformity_glrlm_MYO_ED	-0.13	1.29E-49
Shape	LeastAxis_MYO_ED	-0.11	2.71E-43	Texture	Correlation_glcm_MYO_ES	-0.13	1.26E-48
Shape	Max2DdiameterColumn_MYO_ED	-0.11	1.20E-42	Texture	InformalMeasureofCorrelation1_glcm_MYO_ES	0.12	1.38E-47
Texture	InformalMeasureofCorrelation1_glcm_MYO_ED	-0.11	2.06E-42	Shape	Max2DdiameterRow_RV_ED	-0.12	1.76E-45
Shape	MinorAxis_RV_ED	-0.11	2.32E-42	Texture	InformalMeasureofCorrelation2_glcm_MYO_ES	-0.12	1.97E-45
Shape	Max2DdiameterColumn_RV_ES	-0.11	9.13E-40	Shape	Max2DdiameterColumn_LV_ED	-0.12	6.22E-45
Texture	RunEntropy_glrlm_MYO_ED	0.11	1.42E-39	Shape	SurfaceAreatoVolumeRatio_LV_ED	0.12	6.17E-44
Shape	Volume_RV_ES	-0.11	5.77E-38	Shape	SurfaceAreatoVolumeRatio_LV_ES	0.12	2.26E-43
Shape	LeastAxis_MYO_ES	-0.10	4.77E-37	First order	InterquartileRange_MYO_ED	0.12	4.11E-43
Shape	Max3Ddiameter_RV_ED	-0.10	5.56E-37	Shape	Max2DdiameterSlice_RV_ED	-0.12	5.41E-42
Shape	MinorAxis_LV_ED	-0.10	4.33E-36	Shape	MajorAxis_MYO_ES	-0.12	3.45E-41
Shape	MajorAxis_LV_ED	-0.10	2.06E-35	Shape	Max2DdiameterRow_LV_ED	-0.12	9.97E-41
Shape	Sphericity_LV_ES	-0.10	4.09E-35	First order	RobustMeanAbsoluteDeviation_MYO_ED	0.11	4.70E-40
Shape	SurfaceArea_MYO_ED	-0.10	6.28E-34	Shape	Max2DdiameterColumn_MYO_ED	-0.11	7.40E-40
First order	Median_MYO_ED	0.10	2.22E-33	Shape	MinorAxis_MYO_ED	-0.11	2.80E-38
First order	InterquartileRange_MYO_ED	0.10	4.06E-33	Shape	Max2DdiameterColumn_RV_ES	-0.11	4.66E-38
Texture	InformalMeasureofCorrelation2_glcm_MYO_ED	0.10	8.84E-33	Shape	Max2DdiameterRow_MYO_ED	-0.11	8.27E-38
Shape	Max2DdiameterRow_LV_ED	-0.10	9.41E-33	Shape	SurfaceArea_MYO_ES	-0.11	1.97E-37

Women				Men			
Category	Feature	r	p-value	Category	Feature	r	p-value
Shape	Volume_LV_ES	-0.10	4.32E-32	Shape	MajorAxis_LV_ED	-0.11	3.47E-37
Shape	SurfaceArea_LV_ED	-0.10	5.56E-32	Shape	Max2DdiameterColumn_MYO_ES	-0.11	2.16E-36
First order	Mean_MYO_ED	0.09	4.95E-30	Shape	Max2DdiameterRow_MYO_ES	-0.11	2.90E-36
First order	RootMeanSquared_MYO_ED	0.09	9.30E-30	Shape	SurfaceArea_LV_ED	-0.11	3.59E-36
First order	TotalEnergy_MYO_ED	0.09	1.36E-28	Texture	JointEntropy_glcm_MYO_ED	0.11	3.42E-35
First order	RobustMeanAbsoluteDeviation_MYO_ED	0.09	2.53E-28	Shape	MajorAxis_MYO_ED	-0.10	4.92E-33
First order	InterquartileRange_MYO_ES	-0.09	1.61E-27	Shape	SurfaceAreatoVolumeRatio_RV_ES	0.10	5.92E-33
Shape	LeastAxis_LV_ES	-0.09	6.32E-27	Shape	Max3Ddiameter_LV_ED	-0.10	1.58E-32
First order	Percentile90_MYO_ED	0.09	1.84E-26	Texture	SumEntropy_glcm_MYO_ED	0.10	1.88E-32
Shape	Max3Ddiameter_LV_ED	-0.09	5.49E-25	Texture	InverseDifference_glcm_MYO_ED	-0.10	2.11E-32
Shape	MajorAxis_MYO_ED	-0.09	8.84E-25	First order	Uniformity_MYO_ED	-0.10	2.65E-32
First order	Skewness_MYO_ES	0.08	3.37E-24	Shape	Max2DdiameterRow_LV_ES	-0.10	2.98E-32
First order	Uniformity_MYO_ED	-0.08	5.40E-24	Texture	InverseDifferenceMoment_glcm_MYO_ED	-0.10	3.28E-32
First order	RobustMeanAbsoluteDeviation_MYO_ES	-0.08	1.32E-23	First order	Entropy_MYO_ED	0.10	1.12E-31
Shape	Max2DdiameterRow_LV_ES	-0.08	2.16E-23	Shape	Max2DdiameterRow_RV_ES	-0.10	1.38E-31
Texture	GrayLevelNonUniformityNormalized_glrlm_MYO_ED	-0.08	8.71E-23	Texture	MaximumProbability_glcm_MYO_ED	-0.10	1.85E-31
First order	Energy_MYO_ED	0.08	1.05E-21	Texture	JointEnergy_glcm_MYO_ED	-0.10	2.73E-31
Shape	Max2DdiameterSlice_RV_ED	-0.08	1.20E-21	Texture	DifferenceAverage_glcm_MYO_ED	0.10	2.85E-30
Shape	MajorAxis_MYO_ES	-0.08	2.24E-21	First order	MeanAbsoluteDeviation_MYO_ED	0.10	8.44E-30
Shape	Max2DdiameterColumn_LV_ES	-0.08	4.68E-21	Texture	InverseVariance_glcm_MYO_ED	0.10	1.62E-29
First order	Entropy_MYO_ED	0.08	4.77E-21	Shape	SurfaceAreatoVolumeRatio_RV_ED	0.10	1.67E-29
Texture	Correlation_glcm_MYO_ED	0.08	6.58E-21	Shape	Elongation_RV_ES	-0.10	2.83E-29
Shape	MinorAxis_MYO_ED	-0.08	7.50E-21	First order	Skewness_MYO_ES	0.10	6.85E-29
Shape	Max2DdiameterColumn_MYO_ES	-0.08	1.01E-20	Shape	Max2DdiameterColumn_LV_ES	-0.10	8.06E-29
First order	MeanAbsoluteDeviation_MYO_ED	0.08	8.87E-20	Shape	Max3Ddiameter_RV_ES	-0.10	2.10E-28
Shape	Max2DdiameterRow_RV_ED	-0.08	1.04E-19	Texture	GrayLevelNonUniformityNormalized_glrlm_MYO_ED	-0.10	4.32E-28
Texture	InformalMeasureofCorrelation2_glcm_MYO_ES	-0.08	6.25E-19	Shape	Volume_LV_ES	-0.10	7.21E-28
Shape	Max2DdiameterRow_MYO_ED	-0.07	7.95E-19	Shape	MinorAxis_LV_ED	-0.10	1.25E-27
Texture	Correlation_glcm_MYO_ES	-0.07	1.16E-18	Shape	LeastAxis_LV_ED	-0.10	1.43E-27
Texture	MaximumProbability_glcm_MYO_ED	-0.07	1.48E-18	Shape	Max3Ddiameter_MYO_ED	-0.10	2.10E-27
Texture	SumEntropy_glcm_MYO_ED	0.07	1.73E-18	Texture	RunEntropy_glrlm_MYO_ES	-0.09	2.07E-25
First order	Percentile10_MYO_ED	0.07	2.32E-18	Texture	DependenceEntropy_gldm_MYO_ES	-0.09	1.81E-24
Texture	JointEnergy_glcm_MYO_ED	-0.07	7.41E-18	Shape	Sphericity_MYO_ED	0.09	3.53E-24

Women				Men			
Category	Feature	r	p-value	Category	Feature	r	p-value
Shape	Elongation_RV_ES	-0.07	1.41E-17	Shape	LeastAxis_LV_ES	-0.09	3.59E-24
Shape	SurfaceAreatoVolumeRatio_RV_ES	0.07	1.56E-17	Texture	ZoneVariance_glszm_MYO_ED	-0.09	4.86E-24
Shape	SurfaceArea_LV_ES	-0.07	1.03E-16	Texture	DependenceNonUniformity_gldm_MYO_ES	-0.09	5.00E-24
Shape	SurfaceArea_MYO_ES	-0.07	1.32E-16	Shape	Max3Ddiameter_LV_ES	-0.09	9.24E-24
Texture	GrayLevelNonUniformityNormalized_glszm_MYO_ES	0.07	6.84E-16	Shape	MajorAxis_LV_ES	-0.09	9.44E-24
Texture	GrayLevelNonUniformityNormalized_glrlm_MYO_ES	0.07	1.38E-15	Texture	LargeAreaEmphasis_glszm_MYO_ED	-0.09	1.81E-23
Texture	JointEntropy_glcm_MYO_ED	0.07	1.65E-15	First order	Percentile90_MYO_ED	0.09	1.96E-23
First order	Uniformity_MYO_ES	0.07	2.10E-15	Texture	DifferenceEntropy_glcm_MYO_ED	0.09	7.20E-23
Texture	HighGrayLevelEmphasis_gldm_MYO_ED	0.07	3.24E-14	First order	InterquartileRange_MYO_ES	-0.09	1.35E-22
Shape	LeastAxis_RV_ES	-0.07	5.19E-14	Shape	Max3Ddiameter_MYO_ES	-0.09	6.95E-22
Texture	HighGrayLevelRunEmphasis_glrlm_MYO_ED	0.07	5.22E-14	Shape	Sphericity_MYO_ES	0.09	8.13E-22
Shape	Max3Ddiameter_MYO_ED	-0.06	1.27E-13	Texture	Contrast_glcm_MYO_ED	0.09	1.14E-21
First order	Entropy_MYO_ES	-0.06	1.40E-13	Texture	LargeAreaHighGrayLevelEmphasis_glszm_MYO_ES	-0.08	1.74E-21
Shape	Sphericity_LV_ED	-0.06	1.45E-13	Shape	Max2DdiameterSlice_MYO_ED	-0.08	2.03E-21
First order	Kurtosis_MYO_ES	0.06	1.47E-13	Texture	DependenceNonUniformityNormalized_gldm_MYO_ED	0.08	3.49E-21
Texture	InformalMeasureofCorrelation1_glcm_MYO_ES	0.06	1.55E-13	Texture	SumofSquares_glcm_MYO_ED	0.08	9.59E-21
Texture	LongRunHighGrayLevelEmphasis_glrlm_MYO_ED	0.06	1.65E-13	Texture	RunPercentage_glrlm_MYO_ED	0.08	1.10E-20
Shape	Max3Ddiameter_LV_ES	-0.06	2.77E-13	Shape	MajorAxis_RV_ES	-0.08	1.16E-20
Texture	GrayLevelVariance_gldm_MYO_ED	0.06	4.55E-13	First order	Energy_MYO_ES	-0.08	3.24E-20
First order	Variance_MYO_ED	0.06	6.70E-13	Shape	SurfaceArea_LV_ES	-0.08	5.47E-20
Texture	ShortRunHighGrayLevelEmphasis_glrlm_MYO_ED	0.06	8.57E-13	Texture	LargeDependenceEmphasis_gldm_MYO_ED	-0.08	5.76E-20
First order	MeanAbsoluteDeviation_MYO_ES	-0.06	1.24E-12	Shape	Volume_MYO_ED	-0.08	6.53E-20
Texture	GrayLevelVariance_glrlm_MYO_ED	0.06	1.87E-12	Shape	MinorAxis_MYO_ES	-0.08	3.21E-19
Shape	SurfaceAreatoVolumeRatio_RV_ED	0.06	2.92E-12	First order	RobustMeanAbsoluteDeviation_MYO_ES	-0.08	3.47E-19
Texture	SumEntropy_glcm_MYO_ES	-0.06	3.30E-12	First order	RootMeanSquared_MYO_ED	0.08	4.07E-19
Texture	GrayLevelNonUniformity_glrlm_MYO_ED	-0.06	5.99E-12	Texture	ClusterTendency_glcm_MYO_ED	0.08	8.06E-19
Texture	HighGrayLevelZoneEmphasis_glszm_MYO_ED	0.06	2.17E-11	First order	Kurtosis_MYO_ES	0.08	8.66E-19
Texture	InverseVariance_glcm_MYO_ED	0.06	2.72E-11	Texture	DependenceVariance_gldm_MYO_ED	-0.08	1.12E-18
Texture	GrayLevelNonUniformity_gldm_MYO_ES	0.06	9.00E-11	First order	Median_MYO_ED	0.08	1.77E-18
Texture	GrayLevelNonUniformity_gldm_MYO_ED	-0.06	1.03E-10	Texture	ShortRunEmphasis_glrlm_MYO_ED	0.08	3.13E-18

Women				Men			
Category	Feature	r	p-value	Category	Feature	r	p-value
Shape	Max2DdiameterRow_RV_ES	-0.06	1.11E-10	Texture	RunLengthNonUniformityNormalized_glrlm_MYO_ED	0.08	4.86E-18
Texture	ClusterTendency_glcm_MYO_ED	0.06	2.03E-10	Texture	RunVariance_glrlm_MYO_ED	-0.08	6.14E-18
Texture	GrayLevelVariance_glszm_MYO_ED	0.06	7.36E-10	First order	Mean_MYO_ED	0.08	6.38E-18
Shape	Max2DdiameterRow_MYO_ES	-0.05	1.19E-9	Texture	LargeAreaHighGrayLevelEmphasis_glszm_MYO_ED	-0.08	1.13E-17
Shape	Max3Ddiameter_RV_ES	-0.05	1.30E-9	Texture	GrayLevelVariance_gldm_MYO_ED	0.08	1.29E-17
Texture	Autocorrelation_glcm_MYO_ED	0.05	1.61E-9	Texture	InverseDifferenceNormalized_glcm_MYO_ED	-0.08	1.61E-17
Texture	DependenceEntropy_gldm_MYO_ES	-0.05	1.24E-8	Texture	Contrast_ngtdm_MYO_ED	0.08	2.89E-17
Texture	SumAverage_glcm_MYO_ED	0.05	1.26E-8	Texture	Coarseness_ngtdm_MYO_ED	0.08	3.89E-17
Texture	JointAverage_glcm_MYO_ED	0.05	1.26E-8	First order	Variance_MYO_ED	0.08	9.33E-17
Texture	GrayLevelNonUniformity_glszm_MYO_ES	0.05	1.82E-8	Texture	LongRunEmphasis_glrlm_MYO_ED	-0.07	1.31E-16
Texture	SizeZoneNonUniformity_glszm_MYO_ES	0.05	1.82E-8	Texture	LargeDependenceHighGrayLevelEmphasis_gldm_MYO_ES	-0.07	2.29E-16
Texture	GrayLevelNonUniformity_glrlm_MYO_ES	0.05	2.41E-8	Texture	DependenceEntropy_gldm_MYO_ED	0.07	2.49E-16
Texture	GrayLevelNonUniformity_glszm_MYO_ED	-0.05	4.56E-8	Shape	Volume_MYO_ES	-0.07	8.69E-15
Texture	SizeZoneNonUniformity_glszm_MYO_ED	-0.05	4.56E-8	Texture	ZonePercentage_glszm_MYO_ES	0.07	2.36E-14
Texture	SumofSquares_glcm_MYO_ED	0.05	4.69E-8	Texture	LongRunHighGrayLevelEmphasis_glrlm_MYO_ES	-0.07	3.11E-14
Shape	Flatness_LV_ED	0.05	5.89E-8	Texture	GrayLevelVariance_glrlm_MYO_ED	0.07	4.46E-14
Shape	MajorAxis_LV_ES	-0.05	6.35E-8	First order	TotalEnergy_MYO_ES	-0.07	4.73E-14
Texture	Busyness_ngtdm_MYO_ED	-0.05	1.16E-7	Texture	DifferenceVariance_glcm_MYO_ED	0.07	5.32E-14
Texture	GrayLevelVariance_glszm_MYO_ES	-0.05	1.43E-7	Texture	GrayLevelVariance_glszm_MYO_ED	0.07	2.02E-13
Texture	RunVariance_glrlm_MYO_ES	0.05	2.20E-7	First order	Uniformity_MYO_ES	0.07	3.89E-13
Shape	Max2DdiameterSlice_LV_ED	-0.05	2.29E-7	Shape	Max2DdiameterSlice_RV_ES	-0.06	5.38E-12
Texture	InverseDifference_glcm_MYO_ED	-0.05	2.65E-7	Shape	Max2DdiameterSlice_LV_ED	-0.06	1.41E-11
Shape	MinorAxis_MYO_ES	-0.05	5.45E-7	Texture	GrayLevelNonUniformityNormalized_glrlm_MYO_ES	0.06	1.66E-11
Texture	ZoneEntropy_glszm_MYO_ED	0.05	6.27E-7	Shape	LeastAxis_RV_ES	-0.06	1.71E-11
Texture	LargeDependenceLowGrayLevelEmphasis_gldm_MYO_ES	0.05	7.17E-7	Texture	Complexity_ngtdm_MYO_ED	0.06	3.78E-11
Texture	InverseDifferenceMoment_glcm_MYO_ED	-0.05	9.83E-7	Texture	SmallDependenceEmphasis_gldm_MYO_ES	0.06	4.73E-11
Texture	LowGrayLevelEmphasis_gldm_MYO_ED	-0.05	1.65E-6	Shape	Sphericity_LV_ES	-0.06	4.77E-11
Shape	MinorAxis_LV_ES	-0.05	1.80E-6	Texture	SmallDependenceEmphasis_gldm_MYO_ED	0.06	6.04E-11
Texture	JointEnergy_glcm_MYO_ES	0.05	1.83E-6	Texture	SmallDependenceHighGrayLevelEmphasis_gldm_MYO_ED	0.06	6.56E-11

Women				Men			
Category	Feature	r	p-value	Category	Feature	r	p-value
Texture	Coarseness_ngtdm_MYO_ED	0.05	2.87E-6	Texture	Autocorrelation_glcm_MYO_ES	-0.06	3.17E-10
Texture	LongRunLowGrayLevelEmphasis_glrlm_MYO_ES	0.05	3.21E-6	Texture	SumEntropy_glcm_MYO_ES	-0.06	5.47E-10
Texture	LowGrayLevelRunEmphasis_glrlm_MYO_ED	-0.05	3.33E-6	Texture	JointAverage_glcm_MYO_ES	-0.06	7.83E-10
Texture	SmallAreaHighGrayLevelEmphasis_glszm_MYO_ED	0.04	3.90E-6	Texture	SumAverage_glcm_MYO_ES	-0.06	7.83E-10
Texture	ShortRunLowGrayLevelEmphasis_glrlm_MYO_ED	-0.04	3.94E-6	Texture	SmallDependenceLowGrayLevelEmphasis_gldm_MYO_ES	0.06	1.09E-9
Shape	Max3Ddiameter_MYO_ES	-0.04	4.18E-6	First order	Entropy_MYO_ES	-0.06	3.45E-9
Texture	JointEntropy_glcm_MYO_ES	-0.04	6.28E-6	Texture	ShortRunHighGrayLevelEmphasis_glrlm_MYO_ED	0.06	4.77E-9
Texture	LongRunEmphasis_glrlm_MYO_ES	0.04	6.35E-6	First order	MeanAbsoluteDeviation_MYO_ES	-0.06	4.86E-9
Shape	MajorAxis_RV_ES	-0.04	1.07E-5	Texture	InformalMeasureofCorrelation2_glcm_MYO_ED	0.06	5.30E-9
Shape	LeastAxis_LV_ED	-0.04	1.40E-5	Texture	Strength_ngtdm_MYO_ED	0.06	5.60E-9
Texture	SmallDependenceLowGrayLevelEmphasis_gldm_MYO_ED	-0.04	1.68E-5	Texture	ZonePercentage_glszm_MYO_ED	0.06	5.87E-9
Shape	Sphericity_RV_ED	0.04	2.28E-5	Texture	GrayLevelNonUniformityNormalized_glszm_MYO_ES	0.06	7.76E-9
Texture	Autocorrelation_glcm_MYO_ES	-0.04	2.40E-5	Texture	HighGrayLevelEmphasis_gldm_MYO_ES	-0.05	1.93E-8
Texture	JointAverage_glcm_MYO_ES	-0.04	2.87E-5	First order	Maximum_MYO_ED	0.05	3.48E-8
Texture	SumAverage_glcm_MYO_ES	-0.04	2.87E-5	First order	Range_MYO_ED	0.05	5.10E-8
Texture	DifferenceAverage_glcm_MYO_ED	0.04	3.19E-5	Texture	ZoneEntropy_glszm_MYO_ED	0.05	5.51E-8
First order	Skewness_MYO_ED	-0.04	3.85E-5	Texture	GrayLevelNonUniformity_glszm_MYO_ED	-0.05	3.51E-7
Texture	LargeDependenceLowGrayLevelEmphasis_gldm_MYO_ED	-0.04	4.77E-5	Texture	SizeZoneNonUniformity_glszm_MYO_ED	-0.05	3.51E-7
First order	Median_MYO_ES	-0.04	5.67E-5	Texture	HighGrayLevelRunEmphasis_glrlm_MYO_ES	-0.05	4.56E-7
First order	Maximum_MYO_ED	0.04	9.64E-5	Texture	GrayLevelNonUniformity_glszm_MYO_ES	0.05	7.20E-7
Texture	LongRunLowGrayLevelEmphasis_glrlm_MYO_ED	-0.04	1.81E-4	Texture	SizeZoneNonUniformity_glszm_MYO_ES	0.05	7.20E-7
First order	Range_MYO_ED	0.04	1.92E-4	Texture	Contrast_glcm_MYO_ES	0.05	1.38E-6
Texture	LargeDependenceHighGrayLevelEmphasis_gldm_MYO_ED	0.04	2.10E-4	Shape	Max2DdiameterSlice_MYO_ES	-0.05	3.98E-6
Texture	LargeAreaLowGrayLevelEmphasis_glszm_MYO_ES	0.04	2.55E-4	First order	Median_MYO_ES	-0.05	4.48E-6
Texture	DependenceVariance_gldm_MYO_ES	0.04	2.83E-4	Texture	DifferenceVariance_glcm_MYO_ES	0.05	4.62E-6
Texture	ZoneVariance_glszm_MYO_ES	0.04	3.41E-4	Texture	HighGrayLevelEmphasis_gldm_MYO_ED	0.05	4.65E-6
Shape	Elongation_MYO_ES	0.04	4.89E-4	Texture	LargeDependenceLowGrayLevelEmphasis_gldm_MYO_ED	-0.05	5.93E-6
Shape	Max2DdiameterSlice_MYO_ED	-0.04	4.95E-4	Texture	HighGrayLevelZoneEmphasis_glszm_MYO_ED	0.05	8.85E-6
Texture	RunEntropy_glrlm_MYO_ES	-0.04	5.88E-4	Texture	HighGrayLevelRunEmphasis_glrlm_MYO_ED	0.05	1.20E-5

Women				Men			
Category	Feature	r	p-value	Category	Feature	r	p-value
Texture	Contrast_ngtdm_MYO_ED	0.04	6.45E-4	Texture	LargeAreaLowGrayLevelEmphasis_glszm_MYO_ED	-0.05	1.79E-5
Texture	LargeAreaEmphasis_glszm_MYO_ES	0.04	7.37E-4	Texture	Busyness_ngtdm_MYO_ED	-0.05	2.33E-5
First order	Percentile90_MYO_ES	-0.04	1.01E-3	Texture	LowGrayLevelEmphasis_gldm_MYO_ES	0.04	3.06E-5
Texture	HighGrayLevelEmphasis_gldm_MYO_ES	-0.04	1.06E-3	Texture	ClusterTendency_glcm_MYO_ES	-0.04	3.07E-5
Texture	Complexity_ngtdm_MYO_ED	0.04	1.48E-3	Texture	InverseDifferenceMomentNormalized_glcm_MYO_ED	-0.04	3.35E-5
Texture	GrayLevelNonUniformityNormalized_glszm_MYO_ED	-0.04	1.77E-3	Texture	JointEnergy_glcm_MYO_ES	0.04	4.39E-5
Shape	Volume_MYO_ES	0.03	4.77E-3	First order	Percentile10_MYO_ED	0.04	5.10E-5
Texture	SmallAreaEmphasis_glszm_MYO_ES	-0.03	4.82E-3	First order	Percentile90_MYO_ES	-0.04	6.73E-5
Texture	LargeAreaLowGrayLevelEmphasis_glszm_MYO_ED	-0.03	0.01	Texture	Coarseness_ngtdm_MYO_ES	0.04	6.97E-5
Texture	LargeDependenceEmphasis_gldm_MYO_ES	0.03	0.01	Texture	ShortRunLowGrayLevelEmphasis_glrlm_MYO_ES	0.04	8.00E-5
Texture	SmallDependenceHighGrayLevelEmphasis_gldm_MYO_ED	0.03	0.01	Texture	MaximumProbability_glcm_MYO_ES	0.04	1.47E-4
Texture	MaximumProbability_glcm_MYO_ES	0.03	0.01	Shape	Sphericity_LV_ED	-0.04	1.72E-4
Texture	SizeZoneNonUniformityNormalized_glszm_MYO_ES	-0.03	0.01	Texture	GrayLevelNonUniformityNormalized_glszm_MYO_ED	-0.04	1.75E-4
Texture	LowGrayLevelZoneEmphasis_glszm_MYO_ED	-0.03	0.01	Texture	LowGrayLevelRunEmphasis_glrlm_MYO_ES	0.04	2.67E-4
Texture	Contrast_glcm_MYO_ED	0.03	0.01	Texture	ShortRunEmphasis_glrlm_MYO_ES	0.04	4.14E-4
Texture	ClusterTendency_glcm_MYO_ES	-0.03	0.01	Texture	RunLengthNonUniformityNormalized_glrlm_MYO_ES	0.04	4.37E-4
Texture	HighGrayLevelRunEmphasis_glrlm_MYO_ES	-0.03	0.02	Shape	MinorAxis_LV_ES	-0.04	4.59E-4
First order	RootMeanSquared_MYO_ES	-0.03	0.02	Texture	LargeAreaEmphasis_glszm_MYO_ES	-0.04	5.00E-4
Texture	SmallAreaLowGrayLevelEmphasis_glszm_MYO_ED	-0.03	0.03	Texture	Autocorrelation_glcm_MYO_ED	0.04	5.63E-4
Shape	Flatness_LV_ES	-0.03	0.04	Texture	LongRunLowGrayLevelEmphasis_glrlm_MYO_ED	-0.04	6.05E-4
Texture	Strength_ngtdm_MYO_ED	0.03	0.04	Texture	ZoneVariance_glszm_MYO_ES	-0.04	6.72E-4
First order	Mean_MYO_ES	-0.03	0.04	Texture	LargeDependenceLowGrayLevelEmphasis_gldm_MYO_ES	0.04	1.05E-3
First order	Variance_MYO_ES	-0.03	0.04	First order	RootMeanSquared_MYO_ES	-0.04	1.09E-3
Texture	Busyness_ngtdm_MYO_ES	0.03	0.05	Texture	DifferenceAverage_glcm_MYO_ES	0.04	1.15E-3
First order	Kurtosis_MYO_ED	-0.03	0.09	First order	Mean_MYO_ES	-0.04	1.26E-3
Texture	GrayLevelVariance_gldm_MYO_ES	-0.03	0.09	Texture	DifferenceEntropy_glcm_MYO_ES	0.04	1.63E-3

Women				Men			
Category	Feature	r	p-value	Category	Feature	r	p-value
Texture	DependenceNonUniformityNormalized_gldm_MYO_ED	0.03	0.14	Shape	Flatness_MYO_ED	-0.04	3.32E-3
Texture	LargeDependenceHighGrayLevelEmphasis_gldm_MYO_ES	-0.03	0.17	Texture	SmallAreaHighGrayLevelEmphasis_glszm_MYO_ED	0.04	3.33E-3
Texture	GrayLevelVariance_glrlm_MYO_ES	-0.03	0.20	Texture	SumAverage_glcm_MYO_ED	0.04	6.11E-3
Texture	RunPercentage_glrlm_MYO_ES	-0.03	0.25	Texture	JointAverage_glcm_MYO_ED	0.04	6.11E-3
Texture	ShortRunHighGrayLevelEmphasis_glrlm_MYO_ES	-0.03	0.26	Shape	Elongation_RV_ED	-0.03	9.22E-3
Texture	Coarseness_ngtdm_MYO_ES	-0.03	0.32	Shape	Sphericity_RV_ED	0.03	1.77E-2
Texture	SmallAreaHighGrayLevelEmphasis_glszm_MYO_ES	-0.03	0.38	Shape	Elongation_LV_ES	0.03	1.81E-2
Texture	DifferenceEntropy_glcm_MYO_ED	0.03	0.39	Texture	InverseDifferenceMoment_glcm_MYO_ES	-0.03	2.29E-2
Shape	Sphericity_RV_ES	0.02	0.43	First order	TotalEnergy_MYO_ED	0.03	2.33E-2
Texture	LowGrayLevelEmphasis_gldm_MYO_ES	0.02	0.43	Texture	GrayLevelNonUniformity_glrlm_MYO_ES	-0.03	2.74E-2
Shape	Max2DdiameterSlice_RV_ES	-0.02	0.72	Texture	LongRunLowGrayLevelEmphasis_glrlm_MYO_ES	0.03	3.38E-2
Shape	Elongation_LV_ED	0.01	1.00	Texture	LargeDependenceHighGrayLevelEmphasis_gldm_MYO_ED	-0.03	4.63E-2
Shape	Max2DdiameterSlice_LV_ES	0.00	1.00	Texture	JointEntropy_glcm_MYO_ES	-0.03	4.85E-2
Shape	Elongation_LV_ES	0.00	1.00	Texture	RunEntropy_glrlm_MYO_ED	0.03	5.88E-2
Shape	LeastAxis_RV_ED	0.01	1.00	Texture	InformalMeasureofCorrelation1_glcm_MYO_ED	-0.03	7.11E-2
Shape	Elongation_RV_ED	-0.01	1.00	Texture	InverseDifference_glcm_MYO_ES	-0.03	7.84E-2
Shape	Flatness_RV_ES	-0.02	1.00	Texture	ShortRunHighGrayLevelEmphasis_glrlm_MYO_ES	-0.03	8.19E-2
Shape	Volume_MYO_ED	0.01	1.00	Shape	Elongation_MYO_ES	0.03	0.13
Shape	Elongation_MYO_ED	0.01	1.00	Texture	DependenceNonUniformity_gldm_MYO_ED	-0.03	0.15
Shape	Flatness_MYO_ED	-0.02	1.00	Texture	LowGrayLevelZoneEmphasis_glszm_MYO_ES	0.03	0.20
Shape	Max2DdiameterSlice_MYO_ES	-0.01	1.00	Texture	SmallAreaHighGrayLevelEmphasis_glszm_MYO_ES	-0.03	0.26
Shape	Flatness_MYO_ES	-0.02	1.00	Texture	SmallAreaLowGrayLevelEmphasis_glszm_MYO_ES	0.03	0.29
First order	Minimum_MYO_ED	0.02	1.00	Texture	RunLengthNonUniformity_glrlm_MYO_ES	-0.03	0.36
First order	Energy_MYO_ES	-0.02	1.00	Texture	SmallAreaEmphasis_glszm_MYO_ED	-0.03	0.49
First order	TotalEnergy_MYO_ES	-0.01	1.00	Texture	SmallAreaEmphasis_glszm_MYO_ES	-0.03	0.50
First order	Percentile10_MYO_ES	0.00	1.00	Shape	SurfaceAreatoVolumeRatio_MYO_ES	-0.03	0.50
First order	Maximum_MYO_ES	-0.01	1.00	Texture	Correlation_glcm_MYO_ED	0.03	0.54
First order	Range_MYO_ES	-0.02	1.00	Texture	SizeZoneNonUniformityNormalized_glszm_MYO_ED	-0.03	0.59

Women				Men			
Category	Feature	r	p-value	Category	Feature	r	p-value
Texture	DifferenceVariance_glcm_MYO_ED	0.02	1.00	Texture	GrayLevelVariance_gldm_MYO_ES	-0.03	0.61
Texture	InverseDifferenceMomentNormalized_glcm_MYO_ED	0.01	1.00	Texture	RunPercentage_glrlm_MYO_ES	0.03	0.69
Texture	InverseDifferenceNormalized_glcm_MYO_ED	-0.01	1.00	First order	Variance_MYO_ES	-0.03	0.70
Texture	Contrast_glcm_MYO_ES	0.02	1.00	Texture	SizeZoneNonUniformityNormalized_glszm_MYO_ES	-0.03	0.73
Texture	DifferenceAverage_glcm_MYO_ES	0.00	1.00	Shape	Elongation_LV_ED	0.01	1.00
Texture	DifferenceEntropy_glcm_MYO_ES	0.00	1.00	Shape	Flatness_LV_ED	0.01	1.00
Texture	DifferenceVariance_glcm_MYO_ES	0.01	1.00	Shape	Max2DdiameterSlice_LV_ES	0.00	1.00
Texture	InverseDifferenceMoment_glcm_MYO_ES	0.00	1.00	Shape	Flatness_LV_ES	-0.01	1.00
Texture	InverseDifference_glcm_MYO_ES	0.00	1.00	Shape	LeastAxis_RV_ED	0.01	1.00
Texture	InverseVariance_glcm_MYO_ES	-0.01	1.00	Shape	Sphericity_RV_ES	0.02	1.00
Texture	SumofSquares_glcm_MYO_ES	-0.02	1.00	Shape	Flatness_RV_ES	0.01	1.00
Texture	SmallAreaEmphasis_glszm_MYO_ED	-0.02	1.00	Shape	SurfaceAreatoVolumeRatio_MYO_ED	-0.02	1.00
Texture	LargeAreaEmphasis_glszm_MYO_ED	-0.02	1.00	Shape	Elongation_MYO_ED	-0.02	1.00
Texture	SizeZoneNonUniformityNormalized_glszm_MYO_ED	-0.02	1.00	Shape	Flatness_MYO_ES	-0.02	1.00
Texture	ZonePercentage_glszm_MYO_ED	-0.01	1.00	First order	Energy_MYO_ED	0.01	1.00
Texture	ZoneVariance_glszm_MYO_ED	-0.02	1.00	First order	Minimum_MYO_ED	0.02	1.00
Texture	LargeAreaHighGrayLevelEmphasis_glszm_MYO_ED	0.01	1.00	First order	Skewness_MYO_ED	-0.01	1.00
Texture	ZonePercentage_glszm_MYO_ES	0.00	1.00	First order	Kurtosis_MYO_ED	-0.02	1.00
Texture	ZoneEntropy_glszm_MYO_ES	-0.02	1.00	First order	Percentile10_MYO_ES	-0.01	1.00
Texture	LowGrayLevelZoneEmphasis_glszm_MYO_ES	0.00	1.00	First order	Maximum_MYO_ES	0.01	1.00
Texture	HighGrayLevelZoneEmphasis_glszm_MYO_ES	-0.01	1.00	First order	Range_MYO_ES	0.00	1.00
Texture	SmallAreaLowGrayLevelEmphasis_glszm_MYO_ES	0.00	1.00	Texture	InverseVariance_glcm_MYO_ES	0.01	1.00
Texture	LargeAreaHighGrayLevelEmphasis_glszm_MYO_ES	0.00	1.00	Texture	SumofSquares_glcm_MYO_ES	-0.02	1.00
Texture	ShortRunEmphasis_glrlm_MYO_ED	0.01	1.00	Texture	LowGrayLevelZoneEmphasis_glszm_MYO_ED	-0.01	1.00
Texture	LongRunEmphasis_glrlm_MYO_ED	-0.01	1.00	Texture	SmallAreaLowGrayLevelEmphasis_glszm_MYO_ED	-0.01	1.00
Texture	RunLengthNonUniformity_glrlm_MYO_ED	0.01	1.00	Texture	GrayLevelVariance_glszm_MYO_ES	-0.02	1.00
Texture	RunLengthNonUniformityNormalized_glrlm_MYO_ED	0.01	1.00	Texture	ZoneEntropy_glszm_MYO_ES	-0.02	1.00
Texture	RunPercentage_glrlm_MYO_ED	0.02	1.00	Texture	HighGrayLevelZoneEmphasis_glszm_MYO_ES	-0.02	1.00
Texture	RunVariance_glrlm_MYO_ED	-0.01	1.00	Texture	LargeAreaLowGrayLevelEmphasis_glszm_MYO_ES	0.01	1.00
Texture	ShortRunEmphasis_glrlm_MYO_ES	-0.01	1.00	Texture	RunLengthNonUniformity_glrlm_MYO_ED	-0.02	1.00

Women				Men			
**Category**	**Feature**	**r**	**p-value**	**Category**	**Feature**	**r**	**p-value**
Texture	RunLengthNonUniformity_glrlm_MYO_ES	0.00	1.00	Texture	LowGrayLevelRunEmphasis_glrlm_MYO_ED	-0.02	1.00
Texture	RunLengthNonUniformityNormalized_glrlm_MYO_ES	-0.01	1.00	Texture	ShortRunLowGrayLevelEmphasis_glrlm_MYO_ED	-0.01	1.00
Texture	LowGrayLevelRunEmphasis_glrlm_MYO_ES	0.02	1.00	Texture	LongRunHighGrayLevelEmphasis_glrlm_MYO_ED	-0.02	1.00
Texture	ShortRunLowGrayLevelEmphasis_glrlm_MYO_ES	0.01	1.00	Texture	LongRunEmphasis_glrlm_MYO_ES	-0.01	1.00
Texture	LongRunHighGrayLevelEmphasis_glrlm_MYO_ES	-0.01	1.00	Texture	GrayLevelVariance_glrlm_MYO_ES	-0.02	1.00
Texture	Contrast_ngtdm_MYO_ES	-0.01	1.00	Texture	RunVariance_glrlm_MYO_ES	-0.01	1.00
Texture	Complexity_ngtdm_MYO_ES	0.01	1.00	Texture	Contrast_ngtdm_MYO_ES	0.00	1.00
Texture	SmallDependenceEmphasis_gldm_MYO_ED	-0.01	1.00	Texture	Busyness_ngtdm_MYO_ES	0.00	1.00
Texture	LargeDependenceEmphasis_gldm_MYO_ED	-0.02	1.00	Texture	Complexity_ngtdm_MYO_ES	0.02	1.00
Texture	DependenceNonUniformity_gldm_MYO_ED	0.02	1.00	Texture	LowGrayLevelEmphasis_gldm_MYO_ED	-0.02	1.00
Texture	DependenceVariance_gldm_MYO_ED	-0.02	1.00	Texture	SmallDependenceLowGrayLevelEmphasis_gldm_MYO_ED	0.01	1.00
Texture	SmallDependenceEmphasis_gldm_MYO_ES	-0.01	1.00	Texture	LargeDependenceEmphasis_gldm_MYO_ES	-0.02	1.00
Texture	DependenceNonUniformity_gldm_MYO_ES	0.00	1.00	Texture	GrayLevelNonUniformity_gldm_MYO_ES	-0.02	1.00
Texture	DependenceNonUniformityNormalized_gldm_MYO_ES	-0.01	1.00	Texture	DependenceNonUniformityNormalized_gldm_MYO_ES	0.01	1.00
Texture	SmallDependenceLowGrayLevelEmphasis_gldm_MYO_ES	-0.01	1.00	Texture	DependenceVariance_gldm_MYO_ES	0.02	1.00
Texture	SmallDependenceHighGrayLevelEmphasis_gldm_MYO_ES	-0.01	1.00	Texture	SmallDependenceHighGrayLevelEmphasis_gldm_MYO_ES	0.02	1.00

Supplementary Table 2 footnote. ED: end-diastole; ES: end-systole; GLCM: grey level co-occurrence matrix; GLDM: grey level dependence matrix; GLRLM: grey level run length matrix; GLSZM: grey level size zone matrix; LV: left ventricle; RV: right ventricle; MYO: left ventricular myocardium; NGTDM: neighbouring grey tone difference matrix.

Supplementary Table 3. Significant associations from the PheWAS in exposure categories for men and women

**Primary Demographics**					
Women			Men		
Exposure	r	p-value	Exposure	r	p-value
Average total household income before tax	-0.05	1.15E-9	Number in household	-0.05	2.05-7
Number of vehicles in household	-0.03	2.14E-4	Average total household income before tax	-0.04	2.05E-6
			Number of vehicles in household	-0.04	1.13E-5
			Townsend deprivation index at recruitment	0.04	1.40E-4
			Type of accommodation lived in	0.03	1.10E-3
**Lifestyle and environment**					
Women			Men		
Exposure	r	p-value	Exposure	r	p-value
Number of days/week of vigorous physical activity 10+ minutes	-0.07	5.48E-16	Number of days/week of vigorous physical activity 10+ minutes	-0.13	4.77E-55
Time spent watching television (TV)	0.06	2.28E-13	Number of days/week of moderate physical activity 10+ minutes	-0.08	5.21E-19
Usual walking pace	-0.06	6.74E-12	Time spent watching television (TV)	0.06	3.73E-12
Current tobaro smoking	0.04	4.59E-6	Age stopped smoking	0.12	2.57E-11
Frequency of other exercises in last 4 weeks	-0.06	5.04E-6	Frequency of other exercises in last 4 weeks	-0.08	1.15E-9
Number of days/week of moderate physical activity 10+ minutes	-0.03	1.93E-3	Smoking status	0.05	5.52E-7
Time spent driving	0.03	2.61E-3	Usual walking pace	-0.05	8.00E-7
Bread type	-0.03	4.60E-3	Cereal intake	-0.05	1.30E-6
Pork intake	0.03	4.90E-3	Bread type	-0.05	2.28E-6
Lifetime number of sexual partners	-0.03	6.97E-3	Getting up in morning	-0.04	1.54E-5
Snoring	-0.03	7.00E-3	Duration of vigorous activity	-0.05	1.55E-5
Poultry intake	0.03	7.28E-3	Oily fish intake	-0.04	7.72E-5
Oily fish intake	-0.03	9.01E-3	Duration walking for pleasure	-0.05	1.05E-4
Smoking status	0.03	0.01	Frequency of strenuous sports in last 4 weeks	-0.09	8.46E-4
Average weekly beer plus cider intake	0.04	0.02	Past tobacco smoking	-0.04	1.45E-3
Duration of vigorous activity	-0.04	0.04	Duration of other exercises	-0.05	1.60E-3
Average weekly red wine intake	0.04	0.04	Duration of moderate activity	-0.04	4.37E-3
Cereal intake	-0.03	0.04	Frequency of stair climbing in last 4 weeks	-0.03	0.010
Hot drink temperature	0.03	0.05	Variation in diet	0.03	0.02

			Time spent driving	0.03	0.02
			Difficulty not smoking for 1 day	0.22	0.03
			Average weekly spirits intake	0.03	0.03
			Beef intake	0.03	0.04
			Number of cigarettes previously smoked daily	0.06	0.04
			Average weekly beer plus cider intake	0.03	0.04
			Bread intake	-0.03	0.05
			Current tobacco smoking	0.03	0.05
**Early life factors**					
Women			Men		
Exposure	r	p-value	Exposure	r	p-value
Comparative body size at age 10	-0.07	1.68E-6	Comparative body size at age 10	-0.09	2.10E-8
**Education and employment**					
Women			Men		
Exposure	r	p-value	Exposure	r	p-value
			Job involves shift work	0.05	0.01
			Transport type for commuting to job workplace	-0.05	0.01
**Mental Health**					
Women			Men		
Exposure	r	p-value	Exposure	r	p-value
Health satisfaction	0.06	4.45E-12	Health satisfaction	0.08	1.69E-21
Neuroticism score	0.04	5.65E-5	Frequency of tiredness / lethargy in last 2 weeks	0.05	5.09E-9
Nervous feelings	0.04	2.48E-4	Frequency of unenthusiasm / disinterest in last 2 weeks	0.04	1.69E-4
Worrier / anxious feelings	0.04	3.33E-4	Financial situation satisfaction	0.04	2.94E-4
Financial situation satisfaction	0.03	8.40E-4	Neuroticism score	0.04	1.95E-3
Work/job satisfaction	0.03	1.63E-3	Frequency of tenseness / restlessness in last 2 weeks	0.03	2.00E-3
Frequency of tiredness / lethargy in last 2 weeks	0.03	0.02	Fed-up feelings	0.03	3.41E-3
			Happiness	0.03	4.00E-3
			Nervous feelings	0.03	0.01
			Seen doctor (GP) for nerves, anxiety, tension or depression	0.03	0.01
			Miserableness	0.03	0.02

			Ever unenthusiastic/disinterested for a whole week	0.03	0.02
			Worrier / anxious feelings	0.03	0.034
**Physical measures**					
Women			Men		
Exposure	r	p-value	Exposure	r	p-value
Ventricular rate	0.30	5.80E-284	Ventricular rate	0.31	1.85E-273
Pulse rate, automated reading	0.27	6.31E-214	Pulse rate	0.28	2.33E-223
Pulse rate	0.26	1.58E-206	Pulse rate, automated reading	0.29	3.81E-222
Diastolic blood pressure, automated reading	0.24	6.68E-165	Body fat percentage	0.18	1.07E-98
Waist circumference	0.19	4.94E-121	Whole body fat mass	0.17	9.44E-89
Systolic blood pressure, automated reading	0.19	2.64E-96	Trunk fat percentage	0.17	1.17E-84
Leg fat percentage (right)	0.15	2.20E-73	Trunk fat mass	0.17	7.83E-81
Leg fat percentage (left)	0.14	1.39E-69	Leg fat percentage (right)	0.16	3.77E-80
Body fat percentage	0.13	1.27E-51	Waist circumference	0.16	1.01E-77
Trunk fat percentage	0.11	1.36E-38	Leg fat percentage (left)	0.16	4.88E-76
Whole body fat mass	0.10	1.63E-34	Position of pulse wave notch	0.16	1.43E-73
Arm fat percentage (right)	0.10	3.43E-34	Diastolic blood pressure, automated reading	0.17	1.52E-70
Pulse rate (during blood-pressure measurement)	0.30	4.42E-34	Impedance of whole body	0.14	1.60E-59
Trunk fat mass	0.10	1.00E-32	Position of the shoulder on the pulse waveform	0.15	5.87E-59
Position of the shoulder on the pulse waveform	0.10	8.20E-32	Leg fat mass (right)	0.14	6.49E-59
Arm fat percentage (left)	0.10	1.24E-31	Impedance of leg (right)	0.14	2.64E-56
Leg fat-free mass (right)	-0.10	5.16E-31	Leg fat mass (left)	0.14	4.36E-56
Leg predicted mass (right)	-0.10	7.65E-31	Whole body fat-free mass	-0.14	2.62E-55
Impedance of leg (right)	0.10	8.78E-30	Whole body water mass	-0.14	3.12E-55
Impedance of leg (left)	0.09	1.87E-28	Arm fat percentage (left)	0.14	1.09E-53
Leg predicted mass (left)	-0.09	2.28E-27	Arm fat percentage (right)	0.13	3.20E-53
Leg fat-free mass (left)	-0.09	5.17E-27	Impedance of leg (left)	0.13	6.64E-51
Leg fat mass (right)	0.09	3.21E-25	Trunk fat-free mass	-0.13	2.14E-50
Position of pulse wave notch	0.09	1.21E-23	Trunk predicted mass	-0.13	2.48E-50
Leg fat mass (left)	0.08	2.75E-23	Basal metabolic rate	-0.13	7.70E-47
Diastolic blood pressure, manual reading	0.25	3.12E-22	Leg predicted mass (right)	-0.12	7.81E-44

Position of the pulse wave peak	0.08	9.29E-19	Leg fat-free mass (right)	-0.12	1.18E-43
Pulse wave Arterial Stiffness index	0.08	6.29E-17	Position of the pulse wave peak	0.12	2.58E-36
Whole body water mass	-0.07	2.58E-16	Leg fat-free mass (left)	-0.11	1.77E-32
Whole body fat-free mass	-0.07	2.61E-16	Leg predicted mass (left)	-0.10	2.41E-31
Systolic blood pressure, manual reading	0.20	5.24E-14	Impedance of arm (left)	0.10	8.46E-31
Body mass index (BMI)	0.06	3.12E-13	Impedance of arm (right)	0.10	2.45E-30
Body mass index (BMI)	0.06	4.19E-13	Arm fat-free mass (left)	-0.10	2.69E-30
Impedance of whole body	0.06	1.58E-12	Arm fat-free mass (right)	-0.10	2.87E-30
Basal metabolic rate	-0.06	2.66E-12	Arm predicted mass (left)	-0.10	5.24E-30
Pulse wave peak to peak time	-0.06	1.46E-10	Arm predicted mass (right)	-0.10	2.70E-29
Standing height	-0.06	5.85E-10	Pulse rate (during blood-pressure measurement)	0.29	1.08E-27
Trunk fat-free mass	-0.05	4.03E-9	Arm fat mass (left)	0.09	4.06E-24
Trunk predicted mass	-0.05	7.46E-9	Arm fat mass (right)	0.09	3.85E-22
Forced expiratory volume in 1-second (FEV1), Best measure	-0.06	8.64E-9	Overall acceleration average	-0.12	3.77E-17
Weight	0.05	1.64E-8	Forced expiratory volume in 1-second (FEV1)	-0.08	7.18E-16
Weight	0.05	8.76E-8	Systolic blood pressure, automated reading	0.08	4.00E-15
Forced vital capacity (FVC), Best measure	-0.05	3.82E-7	Forced vital capacity (FVC)	-0.07	4.02E-13
Forced expiratory volume in 1-second (FEV1)	-0.05	3.89E-6	Forced expiratory volume in 1-second (FEV1), Best measure	-0.07	1.45E-12
Forced vital capacity (FVC)	-0.05	4.66E-6	Speed of sound through heel (right)	-0.07	3.93E-12
Hip circumference	-0.04	4.26E-5	Body mass index (BMI)	0.06	1.27E-11
Forced expiratory volume in 1-second (FEV1), predicted percentage	-0.07	4.41E-5	Body mass index (BMI)	0.06	1.32E-11
Overall acceleration average	-0.06	2.91E-4	Weight	0.06	8.13E-11
Maximum heart rate during fitness test	0.09	6.44E-4	Weight	0.06	2.12E-10
Contra-indications for spirometry	0.03	3.16E-3	Heel quantitative ultrasound index (QUI), direct entry (right)	-0.07	2.50E-10
Arm fat-free mass (right)	-0.03	0.02	Heel bone mineral density (BMD) T-score, automated (right)	-0.07	2.50E-10
Peak expiratory flow (PEF)	-0.03	0.03	Heel bone mineral density (BMD) (right)	-0.06	6.10E-10
Hand grip strength (left)	-0.03	0.04	Hand grip strength (left)	-0.06	1.53E-9
			Diastolic blood pressure, manual reading	0.18	1.86E-9
			Minimum carotid IMT (intima-medial thickness) at 240 degrees	-0.06	4.97E-9
			Speed of sound through heel (left)	-0.06	4.15E-8
			Hand grip strength (right)	-0.05	9.80E-8

			Mean carotid IMT (intima-medial thickness) at 240 degrees	-0.05	1.48E-7
			Pulse wave Arterial Stiffness index	0.05	2.26E-7
			Minimum carotid IMT (intima-medial thickness) at 150 degrees	-0.05	4.55E-7
			Heel bone mineral density (BMD) T-score, automated (left)	-0.05	1.22E-6
			Heel quantitative ultrasound index (QUI), direct entry (left)	-0.05	1.22E-6
			Mean carotid IMT (intima-medial thickness) at 150 degrees	-0.05	1.97E-6
			Minimum carotid IMT (intima-medial thickness) at 210 degrees	-0.05	3.19E-6
			Heel broadband ultrasound attenuation (right)	-0.05	4.67E-6
			Heel bone mineral density (BMD) (left)	-0.05	5.52E-6
			Forced expiratory volume in 1-second (FEV1), predicted percentage	-0.08	6.12E-6
			Forced vital capacity (FVC), Best measure	-0.05	1.20E-5
			Minimum carotid IMT (intima-medial thickness) at 120 degrees	-0.05	2.55E-5
			Maximum heart rate during fitness test	0.11	1.02E-4
			Mean carotid IMT (intima-medial thickness) at 210 degrees	-0.04	1.40E-4
			Maximum carotid IMT (intima-medial thickness) at 150 degrees	-0.04	1.41E-4
			Speed of sound through heel	-0.06	1.57E-4
			Peak expiratory flow (PEF)	-0.04	1.60E-4
			Seated height	-0.04	1.78E-4
			Maximum workload during fitness test	-0.10	3.81E-4
			Mean carotid IMT (intima-medial thickness) at 120 degrees	-0.04	4.41E-4
			Maximum carotid IMT (intima-medial thickness) at 240 degrees	-0.04	8.45E-4
			Heel bone mineral density (BMD)	-0.05	8.84E-4
			Heel quantitative ultrasound index (QUI), direct entry	-0.05	9.93E-4
			Heel bone mineral density (BMD) T-score, automated	-0.05	9.94E-4
			Standing height	-0.04	1.02E-3
			Heel broadband ultrasound attenuation (left)	-0.04	1.67E-3
			Volume level set by participant (left)	0.03	0.02
			Maximum carotid IMT (intima-medial thickness) at 210 degrees	-0.03	0.02
			Smoked cigarette or pipe within last hour	0.04	0.04
**Cognitive function**					
Women			Men		

Exposure	r	p-value	Exposure	r	p-value
PM: initial answer	-0.03	1.10E-3	Fluid intelligence score	-0.04	1.65E-3
Fluid intelligence score	-0.03	1.20E-3	Mean time to correctly identify matches	0.03	4.39E-3
Maximum digits remembered correctly	-0.04	0.02	Time to answer	0.03	0.02
FI4 : positional arithmetic	-0.03	0.02			
**Blood biomarkers**					
Women			Men		
Exposure	r	p-value	Exposure	r	p-value
Triglycerides	0.15	1.57E-78	Vitamin D	-0.08	7.55E-19
HDL cholesterol	-0.11	5.88E-36	Triglycerides	0.07	1.42E-15
Apolipoprotein B	0.10	1.93E-35	HDL cholesterol	-0.06	8.02E-10
Sex hormone binding globulin (SHBG)	-0.10	2.19E-27	Alkaline phosphatase	0.05	2.06E-8
Low density lipoprotein (LDL) direct	0.08	7.89E-23	Neutrophil count	0.13	5.22E-8
Urate	0.08	1.16E-18	Gamma glutamyltransferase	0.05	3.35E-7
Cholesterol	0.06	8.40E-12	Alanine aminotransferase	0.05	1.48E-6
Apolipoprotein A	-0.06	5.52E-11	Haemoglobin concentration	0.11	6.66E-6
Neutrophil count	0.14	3.78E-10	SHBG	-0.05	7.66E-6
Glycated haemoglobin (HbA1c)	0.06	1.27E-09	Apolipoprotein B	0.05	7.78E-6
Glucose	0.06	4.91E-09	Glycated haemoglobin (HbA1c)	0.05	1.01E-5
Haemoglobin concentration	0.13	6.08E-09	High light scatter reticulocyte count	0.11	3.16E-5
Reticulocyte count	0.13	1.55E-08	Reticulocyte count	0.11	1.20E-4
White blood cell (leukocyte) count	0.12	4.67E-08	Urate	0.04	1.26E-4
High light scatter reticulocyte count	0.12	6.94E-08	High light scatter reticulocyte percentage	0.10	2.34E-4
Alkaline phosphatase	0.05	1.22E-07	White blood cell (leukocyte) count	0.10	3.34E-4
Haematocrit percentage	0.12	2.36E-07	Haematocrit percentage	0.09	7.15E-4
Gamma glutamyltransferase	0.05	4.32E-07	Reticulocyte percentage	0.10	8.19E-4
Alanine aminotransferase	0.05	1.86E-06	Total protein	0.04	1.29E-3
Red blood cell (erythrocyte) count	0.11	2.29E-06	Cystatin C	0.04	2.03E-3
Reticulocyte percentage	0.11	1.37E-05	C-reactive protein	0.04	2.66E-3
High light scatter reticulocyte percentage	0.10	2.83E-05	Glucose	0.04	3.40E-3
Calcium	0.04	1.02E-4	Urea	-0.03	4.00E-3

Lymphocyte percentage	-0.09	2.04E-4	Lymphocyte percentage	-0.08	0.02
Neutrophill percentage	0.09	4.87E-4	Apolipoprotein A	-0.03	0.04
Creatinine	-0.04	6.99E-4	Neutrophill percentage	0.07	0.04
Total protein	0.04	7.86E-4	Platelet count	0.07	0.04
Albumin	0.04	1.26E-3	LDL direct	0.03	0.05
Mean sphered cell volume	-0.09	1.53E-3			
Direct bilirubin	-0.03	0.01			
Sodium in urine	0.03	0.02			
**Abdominal MRI**					
Women			Men		
Exposure	r	p-value	Exposure	r	p-value
Visceral adipose tissue volume (VAT)	0.29	4.13-89	Visceral adipose tissue volume (VAT)	0.30	2.93E-85
Total trunk fat volume	0.25	1.08E-39	Total thigh fat-free muscle volume	-0.24	4.55E-50
Abdominal subcutaneous adipose tissue volume (ASAT)	0.13	7.60E-19	Total trunk fat volume	0.30	8.84E-49
Proton density fat fraction (PDFF)	0.16	2.62E-13	Total adipose tissue volume	0.25	1.94E-45
Total adipose tissue volume	0.11	6.33E-11	Total lean tissue volume	-0.19	4.36E-28
Total thigh fat-free muscle volume	-0.07	3.00E-5	Posterior thigh fat-free muscle volume (right)	-0.20	5.11E-21
Total lean tissue volume	-0.07	7E-4	Anterior thigh fat-free muscle volume (right)	-0.19	1.79E-19
Posterior thigh fat-free muscle volume (right)	-0.07	2.87E-3	Anterior thigh fat-free muscle volume (left)	-0.19	3.98E-19
Anterior thigh fat-free muscle volume (left)	-0.07	7.89E-3	Posterior thigh fat-free muscle volume (left)	-0.19	4.24E-19
Anterior thigh fat-free muscle volume (right)	-0.07	0.01	Abdominal subcutaneous adipose tissue volume (ASAT)	0.13	3.95E-16
Posterior thigh fat-free muscle volume (left)	-0.06	0.03	Proton density fat fraction (PDFF)	0.11	1.80E-5
**Self reported medical conditions**					
Women			Men		
Exposure	r	p-value	Exposure	r	p-value
Overall health rating	0.08	1.15E-23	Overall health rating	0.11	1.24E-39
Number of treatments/medications taken	0.08	2.89E-19	Number of treatments/medications taken	0.08	7.71E-16
Medication for cholesterol, blood pressure, diabetes, or take exogenous hormones	0.07	7.31E-16	Wheeze or whistling in the chest in last year	0.06	4.27E-12
Medication for pain relief, constipation, heartburn	0.05	8.67E-08	Taking other prescription medications	0.06	3.93E-10
Method of recording time when non-cancer illness first diagnosed	0.05	1.44E-06	Long-standing illness, disability or infirmity	0.06	2.71E-9
Long-standing illness, disability or infirmity	0.04	4.78E-05	Diabetes diagnosed by doctor	0.05	5.91E-8

Diabetes diagnosed by doctor	0.04	4.88E-05	Medication for pain relief, constipation, heartburn	0.04	7.51E-5
Taking other prescription medications	0.04	4.96E-05	Mouth/teeth dental problems	0.04	7.61E-4
Wheeze or whistling in the chest in last year	0.03	3.32E-3	Number of self-reported non-cancer illnesses	0.04	1.62E-3
Mouth/teeth dental problems	0.03	0.01	Shortness of breath walking on level ground	0.03	0.02
Leg pain on walking	0.03	0.01	Fracture resulting from simple fall	0.12	0.02
Chest pain or discomfort	0.03	0.01	Leg pain on walking: action taken	-0.07	0.02
Weight change compared with 1 year ago	0.03	0.03			
Pain type(s) experienced in last month	0.03	0.04			
**Health related outcomes**					
Women			Men		
Exposure	r	p-value	Exposure	r	p-value
Spells in hospital	0.02	0.04	Patient classification on admission (recoded)	0.02	0.05
**ICD10 diagnosis**					
Women			Men		
Exposure	r	p-value	Exposure	r	p-value
Chapter XIX Injury, poisoning and certain other consequences of external causes	-0.03	9.28E-4	Chapter XIII Diseases of the musculoskeletal system and connective tissue	-0.04	5.10E-4
Chapter XI Diseases of the digestive system	0.03	3.59E-3	Chapter XI Diseases of the digestive system	0.03	2.66E-3
Chapter IV Endocrine, nutritional and metabolic diseases	0.03	0.01			
Chapter I Certain infectious and parasitic diseases	0.02	0.05			
**Female-specific**			**Male specific**		
Women			Men		
Exposure	r	p-value	Exposure	r	p-value
Age at first live birth	-0.07	9.50E-12	Number of children fathered	-0.04	6.96E-05
Age at last live birth	-0.05	1.78E-05	Hair/balding pattern	-0.02	0.02

Supplementary Table 3. Results are Pearson correlation coefficients (r) and Bonferroni corrected p-values.

Supplementary Table 4. The number of statistically significant exposure-heart age delta associations observed in men and women (and both) organised by exposure category

Category	Number of exposures in women	Number of exposures in men	Number of exposures common to both
Abdominal MRI	11	11	11
Blood biomarkers	31	28	23
Cognitive function	4	3	1
Education and employment	0	2	-
Early life factors	1	1	1
Female specific factors	2	-	-
Male specific factors	-	2	-
Health related outcomes	1	1	0
ICD10 diagnosis	4	2	1
ICD10 primary death cause	0	0	-
Lifestyle and environment	19	27	13
Mental health	7	13	6
Primary demographics	2	5	2
Physical measures	53	86	49
Self-reported medical conditions	14	12	8
Total	149/736 (20.2%)	193/711 (27.1%)	115/706 (16.2%)

Supplementary Table 4 footnote. Abbreviations: ICD10: international classification of disease 10; MRI: magnetic resonance imaging.
